# When is a career transition successful? a systematic literature review and outlook (1980–2022)

**DOI:** 10.3389/fpsyg.2023.1141202

**Published:** 2023-09-20

**Authors:** Assel Mussagulova, Samuel Chng, Zi An Galvyn Goh, Cheryl J. Tang, Dinithi N. Jayasekara

**Affiliations:** Lee Kuan Yew Centre for Innovative Cities, Singapore University of Technology and Design, Singapore, Singapore

**Keywords:** career transition, career pathway, systematic review, career development, career taxonomy

## Abstract

A definition of career transitions was initially proposed by Louis in 1980. The pace of career transitions has since increased, unraveling traditional linear career pathways. Despite this, we have inadequate knowledge about what defines successful career transitions. Hence, we conducted a systematic review of the scholarship to understand how career transition success is operationalized and to identify research gaps and directions. We identified and reviewed 244 articles published from 1980 to 2022. We found that career transition success outcomes studied fall under the self-referent category, with the outcomes in the other-referent category absent. Further, most studies rely on a mix of objective and subjective success criteria, with no study considering possible interactions between the two. The review revealed a fragmented scholarship of career transition success and an urgency to broaden investigations of career transition success criteria given rapidly evolving employment trends globally.

## Introduction

“Career transitions” is a broad category defined by Louis (1980) as “the period during which an individual is either changing roles (taking on a different objective role) or changing orientation to a role already held (altering a subjective state)” (p. 330). In her seminal article, Louis (1980) discussed the growing prevalence of job and professional changes, and the possible repercussions these changes may have for individuals and managers. More than 40 years later, the pace of career transitions has increased in the face of globalization. Concurrently, the emergence of new digital technologies as part of digital transformations in many organizations and industries globally has brought about new working arrangements, such as portfolio jobs, gig work, freelancing, and so forth. The idea of a “one-life-one-career” (Sarason, 1977) appears to be diminishing (De Vos et al., 2021), while individual career patterns have become increasingly more idiosyncratic (Lyons et al., 2015).

This evolution has been further accelerated by the COVID-19 pandemic as remote working became the norm and individuals globally have started reassessing their work and life priorities. In the short term, we have observed an unprecedented movement of workers in 2021 [the great resignation] (Chugh, 2021), and some forecasts (e.g., Hite and McDonald, 2020; Klotz, 2021) have suggested that this is likely the beginning of a major reorganization of work and individual careers.

Considerable scholarship has been accumulated over the last few decades, covering various forms of career transitions, from organizational entry (Fang et al., 2011; Ellis et al., 2015) and turnover (Bilgili et al., 2017; Hom et al., 2017), to job loss (Van Dierendonck and Jacobs, 2012; Gowan, 2014), expatriate employment (Takeuchi, 2010; Baruch et al., 2016; Kraimer et al., 2016), and retirement (Beehr, 2014; Wang and Shi, 2014), among others. Additionally, different perspectives of career transitions have been considered as well – career transitions as a life stage, adjustment processes during a transition, the decision-making logic underlying transitions, their social embeddedness, and their impact on individual identity (Sullivan and Al Ariss, 2021).

Even then, the literature on what makes a transition a successful event in one’s career (Verbruggen et al., 2015; De Vos et al., 2021) remains fragmented. Up until now, there have been no academic efforts to systematically review the literature and available evidence on successful career transitions, clear up the nomological net and identify the contributing factors of successful career transitions. This oversight has been missing from the conversation on how to prepare individuals for further dramatic changes in the workplace brought about by technological developments and demographic shifts.

In this regard, a recent literature review by Sullivan and Al Ariss (2021) represents a pioneering attempt to make sense of different theoretical perspectives on career transitions. One of the five most prominent theoretical takes on career transitions identified by Sullivan and Al Ariss (2021) is that of adjustment to career transitions. This line of inquiry acknowledges that career transitions are stressful events that upset routines and patterns, introduce uncertainty, and cause discomfort. With this in mind, when, if ever, is a career transition successful? What makes it so?

This literature review aims to increase our knowledge of successful or positive outcomes of career transitions, and their antecedents, as well as to understand the current state of empirical research on successful career transitions. To this end, we conduct a systematic literature review of articles on career transitions published in leading peer-reviewed journals in human resource management and organizational behavior disciplines. The following research questions guide our review: (1) What is a successful career transition and what makes it so? (2) What is the state of the art on successful career transitions? (3) What are the implications for future research in this field and what needs to be explored to advance our knowledge about the successful outcomes of career transitions?

These research questions will be addressed through three specific actions. First, we carry out an analysis of how “successful career transitions” have been conceptualized and instrumentalized, as well as the theoretical bases used in empirical studies to construct and explain their arguments and hypotheses. Second, we establish common antecedents of career transitions deemed successful. Finally, we propose future research directions that will extend the knowledge in this field and provide further information needed to facilitate successful career transitions and prepare workers for increasingly unpredictable career patterns of the future.

This paper contributes to the literature in three different ways. First, it builds on reviews by De Vos et al. (2021), and Sullivan and Al Ariss (2021), by identifying and synthesizing issues of conceptualization, theoretical frameworks, and methodologies related to career transitions. Since the literature to date has shown a large fragmentation regarding career transitions (De Vos et al., 2021; Sullivan and Al Ariss, 2021), this review contributes by increasing and consolidating our knowledge about the types and patterns of career transitions. We establish, that there is no single typology of career transitions. In addition, some types of transitions receive disproportionately more coverage, for example, school-to-work transition, leaving other transitions under-researched.

Second, this study enriches the knowledge of the current state of career transition-related research, including an overview of the relationships between antecedents and successful career transition outcomes. Through the identification of common patterns in career transition success and the contexts and conditions in which career transitions have the most positive outcomes. Thus, using the objective-subjective career transition outcome dichotomy we establish that the research mainly utilizes easily measurable objective outcomes, such as employment status, and salary, while the use of subjective measures remains inconsistent with no attempts to categorize subjective measures into a typology. This has led to difficulty in establishing patterns in antecedents of successful career transitions. We argue that these two challenges stem, in turn, from a lack of a career transition typology that would outline common characteristics and contextual variables for each transition type.

Finally, it identifies research gaps and proposes some promising avenues for future research, which may encourage human resource management academic and professional progress in this area. In particular, it highlights the under-theorized nature of the scholarship on career transitions in general and a lack of an authoritative framework on what constitutes a successful career transition. Further, our analysis of the studies’ profiles demonstrates several important trends: (1) geographical representation heavily skewed towards Western Europe and North America, (2) disproportionate reliance on readily available samples, such as university students, and (3) the dominance of quantitative over qualitative and mixed methods research. These findings, which pertain to both theoretical and methodological challenges, may help to develop new knowledge to better understand career transitions as a general phenomenon.

## Methodology

**Figure 1:**
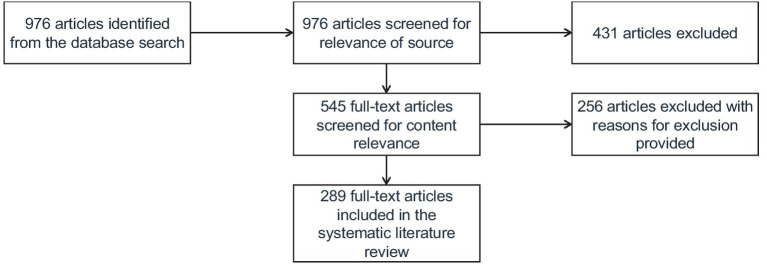
PRISMA flow diagram.

We performed a systematic review adhering to the Preferred Reporting Items for Systematic Reviews and Meta-analyses guidelines to enhance transparency in reporting of our research (PRISMA) (Moher et al., 2009). Refer to Figure 1 for the PRISMA flow diagram.

### Inclusion criteria

Our inclusion criteria are as follow: 1) peer-reviewed journal articles, 2) publication period from 1980 to 2022, 3) journals in organizational behavior and human resource management, and 4) journals in the top 50% of the Scopus CiteScore metric.

This systematic review includes peer-reviewed journal articles, both published in journal issues and those still in press. Our choice to only include peer-reviewed journal articles lies in the general view that they serve as an established source for obtaining novel findings (Ngai et al., 2008). Only empirical articles in English were considered.

Studies published from 1980 to 2022 were considered in the review. The time interval was selected in line with the pioneering conceptualization of career transitions by Louis (1980) and the publication of Schlossberg’s (1981) transition theory. Only articles that address the outcomes and antecedents of career transitions were included.

We only considered journals in organizational behavior and human resource management disciplines. To make an informed selection of publication outlets, we relied on the journal list compiled by Scopus based on the citation data. This includes CiteScore which measures the average citations received per article published in the journal. Sorting journals by their CiteScore metric allows us to compare them within the same field and calculate the percentile into which they fall. We selected the journals which fall under the top 50% of the Scopus list based on the CiteScore metric to ensure the excellence and rigor of the research that we reviewed. This resulted in a list of 120 journals (see Supplementary Appendix A).

### Study selection

The articles were identified in the Scopus database using the broad search term “career transitions,” “career,” and “transitions,” and their derivatives in a title and abstract. Scopus offered 976 articles. Studies were then selected in two phases. The first stage involved scrutiny of articles’ titles and abstracts for relevance. Examining the articles’ titles and abstracts led to 431 articles’ exclusion due to lack of relevance at this stage. The remaining 545 articles were then read in detail to ensure they meet eligibility criteria, with a further 301 being excluded because they did not fit into inclusion criteria. This resulted in a final set of 244 articles that were included in this review to describe successful career transitions and their antecedents (see Supplementary Appendix B).

## Findings: profiles of studies

### Publication trends by year

**Figure 2:**
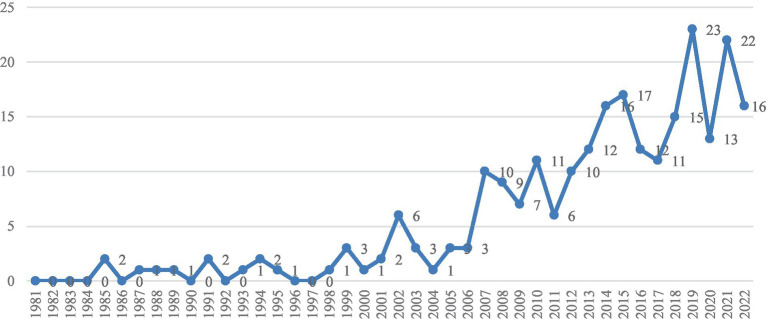
Publication trends by year.

Figure 2 summarizes the number of publications on career transitions between 1981 and 2022. Publications on career transitions only began to emerge in 1985 and there were no relevant publications from 1981 to 1984. There is a marked steady increase in the number of publications on career transitions after 2007. This steady rise in the number of publications parallels the emergence of Savickas (2005) Career Construction Theory (CCT), the protean/boundaryless career orientations (PBCO) framework (Briscoe et al., 2006), and the expansion of social cognitive career theory (Lent et al., 1994). The number of articles published totaled 244, and the highest number of studies published in a year was 23 articles in 2019.

### Publication outlets

Table 1 provides an overview of the journals in which studies in this systematic review were published. For the sake of brevity, Table 1 only reflects journals in which 10 or more articles were published. The Journal of Vocational Behavior was the most frequently selected journal that published relevant articles on career transition research with 56 publications. This is followed by the Journal of Career Development (35), Career Development International (22), Journal of Education and Work (17), Career Development Quarterly (17), Journal of Career Assessment (13), International Journal of Manpower (11), and Work, Employment and Society (11).

**Table 1:** Publication outlets.

Journal	*N* of articles
Journal of Vocational Behavior	56
Journal of Career Development	35
Career Development International	22
Journal of Education and Work	17
Career Development Quarterly	17
Journal of Career Assessment	13
International Journal of Manpower	11
Work, Employment and Society	11

### Geography

Based on the analysis of the geographical distribution of contexts in which career transitions were studied, it was found that 69 (28.3%) studies were conducted in the United States, with the United Kigdom (29; 11.9%) coming in second, and Australia (17; 7%) following. There were also 12 (4.9%) studies studying career transitions in the Netherlands, 12 (4.9%) – in Germany, 12 (4.9%) – in China, 10 (4.1%) - in Finland, and 10 (4.1%) – in Italy. In addition, 10 (4.5%) studies compared two countries or more. In terms of the larger world regions, more than 40% of the studies are set in Europe, and 34% – are in North America, accounting for more than two-thirds of all the studies. East Asia is employed as a study context in 8.6% of the studies and Australia and New Zealand in 8.1% of the studies. Africa, South-East Asia, and South America are especially under-represented, with less than 1% of studies set in each region (Table 2).

**Table 2:** Geographical contexts of studies.

Country/Entity	*N* of articles
USA	69 (28.3%)
UK	29 (11.9%)
Australia	17 (7%)
Germany, Netherlands	12 (4.9%)
China	12 (4.9%)
Finland, Italy	10 (4.5%)
Canada, South Korea	6 (2.4%)
Portugal, Israel	5 (2.3%)
Spain, Switzerland	4 (1.8%)
New Zealand, Hong Kong (P.R.C.), Austria	3 (1.4%)
Turkey, Slovenia, Norway, Japan, Croatia, Belgium	2 (0.9%)
Vietnam, Ukraine, Thailand, Taiwan, Sweden, South Africa, Uzbekistan, Romania, Palestine, Pakistan, Nigeria, Morocco, France, Denmark, Czech Republic, Brazil, Europe, (2 countries): USA and Sweden, Singapore and China, The Netherlands and Greece, (3 countries): USA and Sweden and Germany, Australia and UK and South Africa, Canada and Spain and England (4 countries and more) Hungary and Poland and Lithuania and Slovenia, Belgium and England and The Netherlands and France and Spain and Portugal and Italy and Israel	1 (0.5%)

In addition, we analyzed the country of affiliation of the studies’ authors to understand authorship trends of career transition research (Table 3). As can be seen from Table 3, almost a third of the authors (204; 34.1%) of publications on career transitions are affiliated with institutions in the United States. This is followed by the United Kigdom with 67 (11.1%) authors, Australia (43; 7.2%) and the Netherlands (33; 5.5%), Germany (33; 5.5%), China (32; 5.3%), Finland (27; 4.5%), Italy (25; 4.2%), South Korea (23; 3.8%), and Canada (17; 2.8%). Top regions include North America (221; 36. 9%), Western Europe (158; 26.3%), and East Asia (55, 9.1%). South Korea and China are the only two Asian countries featuring in the top 10 countries by authorship trend, while there are only eight nations in the broader Asian region represented in the entire dataset by authors’ institutional affiliation. The authors note that non-English language journals were not included in this review, and this may contribute to the lower representation of authors from countries where English is not the main language of communication.

**Table 3:** Affiliations of study authors by region.

Country/Entity	*N* of authors
USA	204 (34.1%)
UK	67 (11.1%)
Australia	43 (7.2%)
The Netherlands	33 (5.5%)
Germany	33 (5.5%)
China	32 (5.3%)
Finland	27 (4.5%)
Italy	25 (4.2%)
South Korea	23 (3.8%)
Canada	17 (2.8%)
Belgium	15 (2.5%)
New Zealand	13 (2.1%)
Israel, Portugal	12 (2%)
Austria	10 (1.7%)
Spain	9 (1.5%)
Hong Kong (P.R.C)	8 (1.3%)
Sweden, France, Czech Republic	7 (1.2%)
Switzerland, Norway	6 (1%)
Japan	5 (0.8%)
Croatia	4 (0.7%)
Pakistan, Hungary, Central Asia, Brazil, Nigeria	3 (0.5%)
Turkey, South Africa, Slovenia, Philippines	2 (0.3%)
Ukraine, UAE, Taiwan, Singapore, Ghana, Denmark	1 (0.2%)

### Methodology and data

Out of the reviewed studies, 156 (63.9%) were quantitative, 69 71 (29%) were qualitative, and 15 (6.8%) were mixed methods in nature. In quantitative studies, surveys were the most popular data collection method (122). Qualitative studies mainly relied on interviews (67). Some studies utilized various approaches to secondary data analysis (25). Fifteen studies used a combination of survey and interview methods and 10 studies in the sample employed experimental designs such as quasi-field experiments or randomized control trials. In addition, one study employed open-ended questionnaires or short essays, while one study relied on autoethnography and the diary method, respectively.

Most studies utilized predominantly primary data (213; 87.3%), with secondary data used in 26 studies (10.6%), with two (0.9%) studies using a combination of both. Seventy-eight studies (32%) made use of longitudinal data while 162 studies (66.4%) relied on cross-sectional data.

### Participants

Slightly more than a third of the studies included students as respondents (91, 37.3%) – from middle school to post-graduates. Following this, the second most common sample population was the general workforce (64, 26.2%) commonly derived from population representative panels. Studies that sampled specific career stages such as new hires, early-career individuals, or retirees were the third most common, totaling 39 studies (17.6%). Samples with specific occupations such as athletes, coaches, career counselors and military personnel as well as groups that may face stigma or barriers when entering the workforce both made up 23 or 9.4% of all studies. These groups include (a) persons with special needs and/or persons with disabilities, (b) ex-offenders, and (c) refugees.

## Findings: major theoretical perspectives

Table 4 summarizes the theoretical perspectives most widely used in the studies included in our sample. Some of the most established career theories are the most prevalent in our sample, for example, career construction theory, social cognitive theory, career development theory, and boundaryless careers, among others. Despite the plethora of theories identified, there was a significant variation in the extent to which they were applied. For a sizeable portion of studies, the role of these theories in the studies included in our sample is to shape and justify the use of variables and variable relationships, rather than to theorize what constitutes a successful career transition. In other words, there is next to no emphasis on successful career transitions and what makes them so in using these theories. Instead, they are used to set the scene for the sample and type of transition used, for example, human capital theory (Corrales-Herrero and Rodríguez-Prado, 2014; Evertsson et al., 2015; Galais and Moser, 2018), life transitions (O'connor and Wolfe, 1991; Umney and Kretsos, 2015), portfolio careers (Cohen and Mallon, 1999; Gold and Fraser, 2002; Duberley et al., 2006). Other studies rely on theories to justify their choice of variables, for example, gender theories (Hwang et al., 2018; Mansuy and Werquin, 2018; Ballo, 2020), employability (Lam and Tang, 2020; Ojala et al., 2021; Presti et al., 2021), career construction (Perera and McIlveen, 2014; Šverko and Babarović, 2019; Santilli et al., 2019), and career adaptability (Hui et al., 2018a,b; Pan et al., 2018). Hence, the overarching finding in this regard is that the research on successful career transitions remains under-theorized and requires further theory development and greater theoretical grounding.

**Table 4:** Summary of major theoretical perspectives.

N	Theory name	Author(s) (year)	N of studies
1	Career construction theory	Savickas (2005)	38
2	Career development theory	Super (1957)	12
3	Social cognitive theory	Lent et al. (1994)	12
4	Career transitions	Louis (1980)	9
5	Boundaryless careers	Arthur (1994)	8
6	Identity theory	Ashforth (2000)	7
7	Job mobility	Nicholson and West (1988)	7
8	Life transitions	Schlossberg (1981)	6
9	Gender and social class model	Heppner and Jung (2013)	5
10	Self-determination theory	Deci and Ryan (2000)	5
11	Human capital theory	Becker (1962)	5
12	Goal-setting theory	Locke and Latham (1990)	4
13	Person-job, person-organization, person-environment fit	Kristof (1996)	4 5
14	Portfolio careers	Cawsey et al. (1995)	3
15	Employability	Feintuch (1955)	3
16	Calling	Bellah et al. (1996)	2
17	Career self-management model	Crites (1969, 1976)	2
18	Job demands-resources model	Bakker et al. (2003)	2

There might be two underlying causes for this. First, in the course of our review, we found that there is no single definition of “career transitions” in the academic literature, however, there are several commonly used definitions of career transitions that are prominent in the literature. For example, Louis (1980) defines career transition as the period during which an individual is changing roles or changing their orientation to a role already held; suggesting that career transition is both a process of change and the period during which the change is taking place. Other scholars see career transitions as “events or non-events in the career development process causing changes in the meaning of the career, one’s self-assumptions, and view of the world” (O'Neil et al., 1987, p. 66). Schlossberg’s (2011) definition of career transitions derived from her conceptualization of general life transitions describes this phenomenon as an event, or non-event that results in changed relationships, routines, assumptions, and roles. This lack of a single definition meant that there is no ostensible set of criteria that would be in turn instrumental for producing a set of characteristics of what constitutes a successful outcome of transition.

Second, there is neither a universal typology of career transitions nor a consensus. In addition to providing a definition that is used until this day, Louis (1980) also offered her conceptualization of career transition typology, citing the lack of a helpful framework for understanding and managing career transitions as an important oversight. She proposed that there are five types of career transitions: (1) entering a labor pool, (2) taking on a different role, (3) moving from one organization to another, (4) changing professions or occupations, and (5) leaving a labor pool. Bruce and Scott (1994) in their attempt to revisit and improve upon Louis (1980) typology modified it to entry events, promotion events, lateral moves, resignation, and retirement. In a similar vein, Wanberg and Kammeyer-Mueller (2008) distinguish between five types of major career transitions: (1) initial career choice, (2) organizational entry, (3) job loss, (4) career re-evaluation, and (5) retirement, although they acknowledge that this is not an exhaustive list. Nicholson and West (1988) suggest that there are 12 types of work transitions based on three mobility dimensions: status (upwards, lateral, downwards), function (same or changed), and employer (internal or external). Similar to the definitional scarcity, the studies in our sample could not rely on any career transition typology to describe and categorize the transition in question.

Together, these two findings point to a lack of unifying theoretical approach and significant under-theorization of the field of career transitions. It is, therefore, surprising that to date there has been no attempt to develop an authoritative framework for a successful career transition even though standalone studies attempt to operationalize it but only within the context of one study.

## Findings: empirically tested outcomes and antecedents

The reviewed studies focused on a range of career transition outcomes, as well as a myriad of factors enabling these outcomes. We identified a variety of factors, interventions, and practices that differed in terms of nature, length, intensity, delivery method, and content. Since there are several types of career transitions, which are all distinct from each other, we assumed that the nomological net of successful outcome variables for each transition type should be different. With this in mind, we subdivided this section into findings on outcomes and antecedents, each categorized by type of career transition – based on the typology by Louis (1980) as well as several categories which do not fall neatly into this typology. Louis (1980) proposed that there are five types of career transitions: (1) entering a labor pool, (2) taking on a different role, (3) moving from one organization to another, (4) changing professions or occupations, and (5) leaving a labor pool. Accordingly, we use the following labels based on typology by Louis (1980) when describing career transition types: (1) entry, (2) promotion, (3) lateral movements, (4) resignation, and (5) retirement.

In our attempt to categorize career transition outcomes we relied on the discussion of objective and subjective career outcomes dichotomy by Ruschoff et al. (2021), combined with the nature of criteria for success – self-referent and other-referent (Heslin, 2005). Social comparison theory (Festinger, 1954) posits that individuals tend to compare the outcomes they attain with those of other people, especially where objective evaluation for these outcomes is not available. This reliance on social comparisons with work peers is known as the use of “other-referent success criteria” (Heslin, 2005). Self-referent success criteria, on the other hand, reflect personal standards and references in accordance with a personally defined metric or value.

Table 5 displays a matrix of career transition success outcomes represented in the literature according to these four dimensions with the number of studies in which these outcomes appear specified in parentheses. In the section below we refer to Table 5 throughout our description of the outcomes used in studies on career transitions.

**Table 5:** Four types of career transition success outcomes.

Criteria	Objective	Subjective
Self-referent	employment status (87) salary (13) performance (10) promotion (8) international mobility (1)	*Adjustment & coping (26)* *Affective outcomes:* satisfaction (31) commitment/engagement (12) positive emotional response (8) *Career-related resources:* career adaptability (18) career development (13) job fit/job-qualification fit (11) career decision-making self-efficacy (8) career progression (7) career exploration (7) career construction behaviors (3) career goals/goal pursuit (2) career sustainability (1) job crafting (1) *Job search related resources/actions:* employability/activities (14) job-search self-efficacy (9) career decidedness (5) *Personal resources:* health/well-being (11) growth/learning (6) intrinsic motivation (1) protean career orientation (1) self-efficacy (1) work values (1) *Other:* turnover intention (4) retirement planning (3)
Other-referent	–	–

### Outcomes of transitions

In our reading of the literature in the sample we found no instances of the use of other-referent criteria, either objective or subjective. All the studies in our sample focused only on self-referent criteria for career transition success.

#### Entry into workforce

This is the most prominent career transition type mostly represented by school-to-work transitions with 141 studies in our sample addressing this type of career transition. The most often used outcome in this career transition type is employment status (*N* = 44 48), an objective career transition success outcome which is logical given that some of the more subjective career transition outcomes such as perceptions of work, satisfaction, development, and progression are warranted. This is followed by an umbrella outcome of “satisfaction” (*N* = 16) which encompasses subjective contentment with various aspects of the job, such as salary, work conditions, leadership, as well as overall job satisfaction and life satisfaction. Adjustment and coping, career adaptability, and employability (*N* = 12 each) are the next most common school-to-work transition outcomes. Adjustment is an important perspective that acknowledges the volatile and stress-inducing nature of career transitions, even expected ones like school-to-work transition (Lipshits-Braziler et al., 2018; Pham and Murray, 2018; Chamandy and Gaudreau, 2019). Career adaptability is defined as “readiness to cope with changing work and work conditions” (Savickas, 1994, p. 58), as well as employability, which refers to a broad range of factors contributing to a person’s ability to secure paid work (De Vos et al., 2021), are widely studied in the context of career transitions. Another commonly used operationalization of successful outcomes of school to work transition is job fit and job-qualification fit (*N* = 9) which are seen as crucial for future positive work outcomes (Jackson, 2014; Neuenschwander and Hofmann, 2021). Another objective career transition outcome used in the studies in our sample is salary (*N* = 7). Career development, career decision-making self-efficacy, health and well-being, and job search self-efficacy were used in 7 studies each. Career decidedness and positive emotional response are less common (*N* = 4 each), while the rest of the outcomes are addressed in two studies or less. We noted that despite the rise of publications on protean and boundaryless career orientations following the Briscoe et al. (2006) article, only one study chose to operationalize a successful school to work transition outcome as protean career orientation as we will discuss further.

#### Promotion

Promotion as a type of career transition was markedly less studied in our sample with only 15 studies focusing on it. We identified three subtypes of promotions: within organizations (intraorganizational); between organizations (inter-organizational); and between industries or sectors (intersectoral). Each of these three subtypes relies on different conceptualizations of a positive outcome of transition. Intraorganizational promotion was the most common transition studied with 4 studies looking at upward mobility (Rigotti et al., 2014; Ruiz, 2014; Day, 2015; Evertsson et al., 2015); 3 studies focusing on employment status (Ulrich and Brott, 2005; Kattenbach et al., 2014; Cappellini et al., 2019); and 2 studies each on satisfaction (Rigotti et al., 2014; Zhou et al., 2021), and adjustment and coping (Oplatka, 2001; Manderscheid and Harrower, 2016). Other outcomes were covered in single studies, including commitment, career decision-making self-efficacy, learning, learning, well-being, performance, and salary (Chudzikowski, 2012). Less well-studied was an inter-organizational promotion where we identified 2 studies that looked at commitment (Thompson and Van de Ven, 2002), and performance (Bruce and Scott, 1994). Intersectoral promotion was the least studied and we identified only one study that did so with career development as the outcome (López‐Andreu et al., 2013).

#### Lateral moves

This category of career transitions refers to hierarchically and sometimes functionally equivalent positions within different contexts – departments, organizations, sectors, countries, and so forth (Campion et al., 2021). In our sample, 45 studies looked at lateral moves and they can be organized into four subtypes: within the same department or organization (inter-role); between organizations (inter-organizational); between sectors (intersectoral), and overseas. The most common outcome for inter-role lateral moves are adjustment and coping (2); career development (2); career progression (2); employment status (2); growth and learning (2); and satisfaction (2). To characterize outcomes of inter-organizational lateral moves researchers most often chose employment status (6), and adjustment and coping (2). Employment status was also the most commonly used outcome for intersectoral lateral moves (4), followed by satisfaction (3). Similarly, employment and status (3), and adjustment and coping (2) were used to operationalize transition outcomes in lateral career moves abroad. Other types of outcomes for all types of lateral moves were used in single studies only, emphasizing the scarcity of research on lateral move transitions.

#### Resignation

Resignation was the least studied career transition type. Only two studies focused on resignation and between them, they investigated three outcomes: engagement, performance, and positive emotional response (Stout et al., 1987; Forret et al., 2010).

#### Retirement

Leaving the workforce due to reaching retirement age was addressed in 13 studies in our sample. The majority of these studies chose to operationalize an outcome of retirement as employment status (8 studies), as most of these studies focused on bridge employment or career renaissance (Cahill et al., 2017; Lahlouh et al., 2019; MacKenzie and Marks, 2019). Two studies looked at retirement planning, one study – at retirement intention, and one study each – at career progression, commitment, performance, and satisfaction.

#### Other transition types

The remainder of the studies either did not mention the specific type of career transition they were addressing (17 studies), or the type of transition did not fall neatly into one of the five categories conceptualized by Louis (1980) in her typology (29 studies). Table 5. is a summary of career transition types in our sample that do not match the criteria of the Louis (1980) typology. Involuntary transitions and transitions from school to higher education are the most commonly addressed in this group of studies, followed by transitions from full-time to part-time jobs, to self-employment, and to portfolio careers, in which individuals develop a portfolio of skills that they sell to a range of clients (Cawsey et al., 1995). Other types include work to school, part-time to full-time employment, precarious to stable employment, and stalled career.

Table 6 summarizes successful career transition outcomes for these transition types. Employment status is the most commonly used career transition outcome across the different transition types, with performance, adjustment, and coping used in other studies. Overall, there seems to be no general trend of relying on a particular outcome more than others, except for employment status.

**Table 6:** Transition outcomes outside of Louis (1980) typology.

Career transition types outside of Louis (1980) typology	*N* of studies in the sample
Involuntary	7
School → higher education	4
Full-time → part-time	4
To self-employment	3
To portfolio career	2
Work to school	2
Part-time → full-time	1
Precarious → stable	1
Stalled	7

### Antecedents of transitions

The picture on antecedents is much less straightforward as there is more variety in the choice of antecedents than in outcomes. Therefore, a difficulty arose in trying to categorize antecedents and analyze them by groups. Table 7 presents antecedents by transition type and outcome of transition. The most prominent antecedent, mentioned in seven types of career transitions, is demographic characteristics, including gender, race, and age. This is followed by social support which was used as an antecedent in studies of six types of transitions. Participation in career programs and interventions was prominently featured in entry career transitions, such as school to work, while career orientations, career self-management, and reliance on career counseling were more commonly used in studies of lateral movements. Other antecedents in our sample of studies pertained to various aspects of career self-management and career development. While it is challenging to establish a pattern due to the heavy fragmentation of antecedents, the overall picture suggests that the majority of antecedents are individual, rather than contextual. In other words, most researchers use antecedents that are in an individual’s control rather than external to the individual undergoing a career transition.

**Table 7:** Summary of successful transition antecedents by transition type.

Prominent career outcomes				
Career stage	Type of transition	Job search related resources/actions	Employment status	Personal resources
Early career	Entry	A) Individual perceptions relating to career -Individual perceptions of skills and employability -Career self-efficacy -Career insight Career planning B) Advantages and Disadvantages related to career -Career barriers -Access to resources C) Experiences -Career programs -Internships -Professional -Experience -Voluntary work D) Programs and Individual Actions -Social support -Career counselling -Life design group intervention -Productive coping strategies	A) Individual perceptions and actions relating to career -Career adaptability -Career adaptive responses -Job search strategies -Labour market ambiguity B) Individual attributes and access to resources -Demographic characteristics -Access to resources -Social capital Social connections -Social support -Social networks -Personal capital -Professional experience	A) Demographic characteristics B) Individual perceptions -Compromise -Attachment anxiety -Perceived Parenting style C) Individual actions -Achievement -Learning D) Career adaptability E) Organizational characteristics
Mid career – upskilling or continued education	Work to school			
	School to higher education		A) Individual characteristics -Demographic characteristics -Gender -Social position -Family characteristics -School performance B) Attitudes -Career -Engagement -Satisfaction	-Demographic characteristics; -Training
Mid career – progression or positive change	Promotion		-Demographic characteristics -Skills -Training -economic environment -Flexibility	-Type of transition -Transition characteristics
	Lateral		A) Objective experience -Skill utilization; -Career programs -Organizational support B) Individual perceptions and traits -Attitudes towards work values and practices -Career orientations -Community engagement -Cultural capital -Social capital -Vocational pride	-Personal calling
Mid career –change in work arrangement	Full-time to part-time			
	To self-employment		-Personality traits -Family characteristics -Mentorship	
	Part-time to full-time		-Demographic characteristics -Education -Motivation -Priorities social skills	
Mid career –change in industry/field	Precarious to stable		-Socioeconomic background	
	To portfolio career			
Mid career – unforeseen or negative change	Involuntary	-Career orientations -Career self-management -Mobility -Career counselling	-Career orientations -Career self-management -Demographic characteristics -Hiatus -Mobility -Social support	
	Stalled			
	Resignation			
Late career	Retirement		A) Individual Characteristics -Demographic characteristics -Education -Financial situation -Health B) Individual characteristics relating to work -Person-vocation fit -Person-job fit -Person-organization fit -Person-group fit -Social support -Organizational change	
	Non-specific transition		-Demographic characteristics -Professional experience -Network -Perceptions of work context -Safety net	

Prominent career outcomes				
Career stage	Type of transition	Adjustment & coping	Career-related resources	Affective outcomes
Early career	Entry	A) Individual perceptions -Belonging -Perceived discrimination -Career doubt -Locus of control B) Actions and resources -Career skills -Productive coping strategies -Goal appraisal C) Programs and policies -Guidance -Career programs -Training	A) Demographic characteristics -Gender -Demographic Characteristics -Demographic variables B) Individual characteristics and perceptions about themselves -Personality traits -Self-esteem -Self-efficacy -Self-knowledge C) Values/ attitudes -Career-related filial piety -Cultural beliefs -Norms and values -Expectations D) Individual actions -Personal exploration -Learning -Goal appraisal -Construction exercise E) Support sources -Mentorship -Career support -Social support F) Individual approaches and perceptions relating specifically to careers -Career thoughts -Career planning -Career decidedness -Career decision-making -Career maturity -Efficacy G) Programs/Jobs -Career development programs -Training -Group career Internships -Internships -Job	A) Objective aspects of job -Salary -Job characteristics B) Individual attitudes and perceptions relating to job/career -Job fit -Motivation -Work values -Career maturity
Mid career – upskilling or continued education	Work to school		A) Personality traits -Commitment to learning -Ego development -Inner directedness B) Experience -Progression -Scope of transition	
	School to higher education	-Optimism		
Mid career – progression or positive change	Promotion	-Self-concept -Decision Making -Learning -Collaboration -Social support	A) Experience at work -Promotion -Training -Working conditions -Organizational characteristics B) Demographic characteristics	-Type of transition
	Lateral	-Mentorship -Career capital -Social capital -Cultural capital -Diversity -Network	A) Individual Characteristics and perceptions -Perceived barriers -Financial situation; B) Experience at work -Working conditions -PROMOTION -Professional growth -Training -Social support -Support systems	-Type of transition
Mid career –change in work arrangement	Full-time to part-time	-Identity		
	To self-employment			
	Part-time to full-time	-Training		
Mid career –change in industry/field	Precarious to stable			
	To portfolio career		-Network -Norms -Skills	
Mid career – unforeseen or negative change	Involuntary			-Organizational enabling characteristics
	Stalled		-Demographic characteristics	
	Resignation			-Type of transition
Late career	Retirement			-Type of transition
	Non-specific transition	-Demographic characteristics -Career counselling	-Social support -Work values -Demographic characteristics	-Scope of transition

## Discussion

In this section, we will discuss several theoretical gaps and opportunities for further research identified as a result of this systematic literature review. We will also consider how practical implications and recommendations developed by researchers in their studies can benefit practitioners seeking policy solutions aimed at supporting individuals undergoing various career transitions.

### What is a successful career transition?

Scholars generally agree that while there are features unique to different types of career transitions, they share similarities as well, particularly, change, uncertainty, and disruption of familiar routines. What does this mean for an individual undergoing a career transition? In organizational psychology, the adjustment phase after (intra- and inter-organizational) career transitions is one focal point (Latack, 1984; Nicholson, 1984). A recent literature review by Sullivan and Al Ariss (2021) attempts to make sense of different theoretical perspectives on career transitions, one of which is the adjustment to career transitions. Their summary of the literature on the adjustment perspective of career transitions (Nicholson and West, 1988; Callister et al., 1999; Eby and Dematteo, 2000) acknowledges that this is essentially a stress-coping perspective: career transitions are often stressful because they entail uncertainty and change, disrupt patterns and routines, and may threaten people’s self-concept (Pinder and Schroeder, 1987; West et al., 1987; Moyle and Parkes, 1999; Armstrong-Stassen, 2003).

Based on our review, we identified two important implications for research on career transitions. First, our findings support Wanberg and Kammeyer-Mueller’s (2008) critique that there have been no recent attempts to revise Louis (1980) typology in line with emerging types of career transitions such as those from full-time employment to freelancing, self-employment, portfolio work, as well as transitions from work to further studies, transition to part-time employment and so forth. Hence, there is an urgent need for a definitive framework for categorizing career transitions that incorporate recent developments in this area to inform and spur further career transition research, especially on ways to manage career transitions brought about by increasingly shorter cycles of technology introduction and reorganization of work accelerated by the COVID-19 pandemic.

Second, research in this area should move towards adopting a less pathological view of career transitions. Most studies identified in this review view career transitions as necessitating adjustment rather than a process that can result in positive or successful outcomes. For example, some organizational psychology researchers examine the positive impact of career transitions on organizational commitment, satisfaction, and engagement (Kalleberg and Mastekaasa, 2001; Gesthuizen and Dagevos, 2008; Verbruggen et al., 2015). These, however, are few and far between. Several studies that explicitly mention “successful” career transitions fail to define what that means precisely and call for further research in this direction (De Vos et al., 2021). Others define them narrowly without developing a theoretical perspective on what entails a “successful” career transition or what makes it such. While we acknowledge the often-subjective nature of “success” we wonder what characteristics would “successful” career transitions bear.

It is not therefore surprising that to date there is no definition of a successful career transition or a detailed characterization of the distinct features associated with a successful transition. Authors who conceptualize transitions as “successful” in their research narrowly define the metrics of success within the limits of their studies and for those studies only. These metrics do not necessarily apply in the real world or across different contexts.

Most authors use a simplistic conceptualization of a successful transition as defined by having attained employment (Bynner and Parsons, 2002). However, as Ruschoff et al. (2021) argue, merely finding employment may no longer be an adequate indicator of a successful transition. This is due to the increasingly fractured nature of employment experiences, especially at the early career stages which are characterized by instability and temporary outcomes, with people exploring different types of work (Vuolo et al., 2012) and proactively seeking to optimize their transition and career outcomes (De Vos et al., 2019) beyond the attainment of a job. Another argument often employed in favor of moving beyond measuring career transition outcomes with employment status is the need to factor in individual preferences and motivations for transitions. This explains the choice to operationalize successful career transitions as the perceived correspondence between employment and career wishes (Hirschi, 2010; Vuolo et al., 2012), satisfaction with employment (Hirschi, 2010), or trajectories of life satisfaction (Ranta et al., 2013; Upadyaya and Salmela-Aro, 2017). Organizational psychology researchers also started to examine subjective outcomes of career transitions, such as the impact on organizational commitment, satisfaction, and engagement (Kalleberg and Mastekaasa, 2001; Gesthuizen and Dagevos, 2008; Verbruggen et al., 2015).

Even less is known about the factors and circumstances that make career transitions successful. Again, this could be attributed to the lack of an overarching framework as well as a consensus on the definition and typology of career transitions and “successful career transitions,” in particular. The literature is fragmented with the bulk of the studies exploring antecedents of a few types of career transitions, for example, school-to-work transitions in vocational psychology and inter-organizational transitions in organizational psychology. These include both individual and environmental factors, such as self-efficacy, goal setting, and preparedness (Feather and O'Brien, 1986; Lent et al., 1994, 2008; Saks, 1995), developmental issues (Bynner, 1997; Savickas, 1999), family influences (Blustein et al., 2002; Settersten, 2005; Ling and O'Brien, 2013), and school performance and education (Saks and Ashforth, 1999; Coles, 2000; Pinquart et al., 2003). In our sample of studies, we find that most antecedents are individual, rather than exploring the role of the environment. It could be argued that this finding can be interpreted as a challenge – more needs to be known about the role of the environment to design more focused policies. At the same time, it presents an opportunity to focus on what is in individual control when it comes to enabling successful career transitions.

### What is the state of the art on successful career transitions?

First, the overwhelming majority of papers in our study are set in countries in Northern and Western Europe, North America, as well as Australia. This could reflect differences in academic traditions, research capabilities, endowments, as well as priority areas for research. While East Asian countries are represented in the sample, research on other regions is still less common. Africa, South-East Asia, and South America are rarely used as study contexts, with even less research on Russia and Central Asia. These regions house rapidly developing economies where swift changes in industries and the organization of work are anticipated. The prevalence of findings from Western contexts in shaping theory and future research agenda, while understandable in the early years of research on career transitions, may be preventing a variety of perspectives from emerging and a multitude of the country- and culture-specific factors from being considered. For example, women’s careers might take different trajectories depending on the country’s context. School-to-work transitions may follow different patterns according to the state of the economy, the dominant sector of employment, attitudes toward vocational qualifications, among others.

In this regard, relatively few comparative studies in the field of career transitions may signify another opportunity to venture into hitherto unexplored geographic, cultural, and social contexts. This could help further the theory by testing its external validity.

Second, the reliance of researchers on students as a research sample can be indicative of the difficulties in obtaining access to other samples, or the keen interest of researchers in school-to-work transition as the first career transition in an individual’s career. In any case, the current research landscape on successful career transitions is heavily skewed towards using university students as a sample of choice, and this, in turn, informs the overall choice of the transition type: school-to-work transition is the most commonly used transition example in our sample of studies.

Third, Louis typology developed in 1980 needs revisiting and updating in accordance with the latest trends in the workforce movements. The rise of the gig economy signifies a seismic shift in the way people see their careers and career development. The COVID-19 pandemic resulted in many people losing their jobs or having their careers suffer a setback with a need to reinvent themselves or transition into a completely different profession.

In this regard, the need for learning new skills (i.e., upskilling, retraining) as part of one’s career journey is a common theme in career research (Shirani, 2019; Monear, 2020), however, based on our findings, the current research landscape on career transitions has yet to catch up with these latest developments. It is important to note that we included articles in the press in our analysis, meaning that there might be yet unpublished research on career transition outcomes in the light of the pandemic in the process of being published.

Fourth, research on successful outcomes of career transitions exists, but far from theorizing on the framework for enabling career transition success, most researchers stop at instrumentalizing a certain outcome as a “successful career transition” without venturing into theory. The objective versus subjective career transition outcome dichotomy is still relevant, however, a more authoritative framework may be needed that would include various contextual, multi-level variables. This would allow for a composite and realistic approach to career transition success. In addition, we note that some variables are used as both antecedents and outcomes. For example, job satisfaction has been used as both. Low job satisfaction can serve as an impetus for a career transition (Nielsen, 2016; Ruiz-Castro et al., 2020), and success of a career transition can be judged based on the attainment of higher job satisfaction in the new job (Xu, 2016; Zhou et al., 2021). Future research could explore how to incorporate antecedents into a unified typology of outcomes.

Fifth, experiences of groups that face barriers (persons with special needs or disabilities, ex-offenders, refugees) are underexplored in the literature and accounted for less than 10% of papers in our sample. Common problems faced were difficulties finding and entering a new job, low wages, limited vocational options, among several other barriers. In our view, current theories and approaches may not sufficiently account for such career progression pathways.

Overall, over the past 43 years, studies on career transitions have undergone significant changes, reflecting the broader changes in the world of work and advancements in research methodologies. There is greater recognition of the importance of career transitions and the non-linear nature of careers given the changing nature of work and the increasing frequency with which individuals change jobs and careers.

Furthermore, previously, career transitions were typically thought of as moving from one job or profession to another. However, the definition of career transitions has broadened to include various other types of career changes, such as returning to work after a career break, transitioning from full-time to part-time work, or changing career roles within an organization. More attention is paid to the psychological and emotional aspects of career transitions, their impact on individuals, including the stress and uncertainty that come with changing careers or losing a job.

### What are the implications for future research?

The implications for future research are informed by three groups of findings from our literature review: theoretical approaches, operationalization of outcomes, and profile of studies.

First, when it comes to a definition and typology of career transitions, there is no one authoritative framework that would be used as a guiding approach to studying various forms of career transitions. Some studies in our sample did not even provide a definition of a career transition or discuss its type and how it would inform the variables and the variable relationship operationalization. This, in turn, explains why no studies defined a successful career transition or even attempted to describe several common criteria. One study (Zikic et al., 2010) discussed the dichotomy of successful career transition characteristics along the objective-subjective line without expanding it further or providing a discussion of the contexts in which one of the two is more appropriate. Employment status may be chosen as an outcome variable for studying successful career transitions as the easiest variable to operationalize career transition outcomes. Most studies relying on this outcome type fail to discuss that employment status alone may not be sufficient to measure the success of a career transition as individuals perceive it.

Future research could address this theoretical void and focus on developing an understanding of the factors that make career transitions successful not only in terms of objective and subjective success but important criteria within each that contribute to perceptions of career transitions as successful experiences. In this regard, the Career Transitions Inventory (Heppner, 1998) is a useful instrument that empirically tested several subdimensions of a positive career transition experience. The ambition of the researchers in this area could turn to reverse-engineer a sound theoretical approach toward these subdimensions.

Second, Tables 5, 6 in this article are clear indications that some career transitions and outcomes are studied more than others. This could be a function of how interesting those career transitions are to the researchers as well as how crucial the findings are for the cohort of individuals undergoing those transitions. On the other hand, this could also be a consequence of opportunist research choices. For example, entry career transitions, a disproportionate amount of which are school-to-work transitions, might be so widely studied as students are often one of the most accessible samples to researchers.

In the current environment, research opportunities are plenty – in the face of the Great Resignation induced by the global pandemic new avenues will potentially open up for studying other types of transition which remain blank (Table 5), such as various forms of promotion and lateral moves, as well as transition types which do not fall neatly into the existing typologies, such as part-time employment, gig work, return to education, self-employment and persons that face barriers such as persons with special needs or disabilities, ex-offenders, or refugees. There will likely be a need to develop new theories and approaches to understand the experiences of these individuals who have to undergo career transitions either by choice or circumstance.

Third, profiles of studies are indicative of the overall dominance of research hailing from North America and Western Europe. We acknowledge that this might be due to our choice to only include the publications in the English language, however, our search of the Scopus database yielded only a few abstracts published in other languages (such as French, Spanish, Korean, Mandarin, etc.). This presents an opportunity for researchers from other world regions to expand the scholarship on career transitions to hitherto unexplored geographical, city, cultural, and economic stages of development settings to better understand whether the conceptual foundations still hold.

In addition, a need for a greater diversity of methodological approaches and data types may be evident. More research relying on quasi-experimental designs would enable a more robust conceptualization of variable relationships while utilizing qualitative and mixed-method research methods may be instrumental for bringing in a humanistic perspective and developing an in-depth understanding of individual transition experiences.

## Conclusion

This research provides a review of what we know to date about successful career transitions and how we could usefully develop this topic in the coming years. It also identifies some patterns in career transition research. As careers are becoming less linear and the changing work arrangements and modes are inevitable due to the shockwaves sent by the pandemic, the review is timely and provides a comprehensive picture of how scholars have defined and studied successful career transitions, the main methodological considerations, the main areas of research related to successful career transitions (antecedents and outcomes), and the overall state of the art. Based on this, some insights and guidelines for future research have also been provided. In summary, based on the answer to our four research questions, this review can provide academics with specific knowledge about successful career transition antecedents and outcomes, outlining interesting research possibilities for developing the field.

Our contributions also provide valuable suggestions for human resource management (HRM) practitioners. Firstly, HRM can facilitate the transition process of new hires by acknowledging different types of career transitions and their outcomes, as well as factors enabling these outcomes. Second, companies should also be aware of individual differences in work priorities, for example by age group (Wrzesniewski et al., 2013; Kuron et al., 2015). Thirdly, successful outcomes of career transitions may be facilitated through enhancement strategies for individuals who are about to make a career transition (Lavallee, 2006). This includes identifying skills that they had developed in their previous careers that could be transferred to other areas of their lives (e.g., leadership, communication, and performing under pressure). Finally, Van der Horst et al. (2017) emphasize that creating a stimulating work environment and fostering an internal locus of control, generalized self-efficacy and career curiosity are key in helping workers maintain adaptive responses throughout their careers. These could be fostered by including exercises to promote exploration in formal learning settings and informal learning contexts through supportive organizational policies and procedures (Reio and Wiswell, 2000).

Despite our efforts to identify the publications to include in this review to provide a comprehensive picture of successful career transitions and to adopt a rigorous methodology and approach to the analysis of these papers, there are several limitations. First, our literature search may not have captured all the sources that address the subject of this review since we only included sources that explicitly mentioned “career transitions” as well as those that were indexed in Scopus and have undergone extensive peer review. Therefore, this review may suffer from a general limitation of systematic literature reviews, which is the exclusion of relevant studies, conference papers, and book chapters due to the rigorous inclusion and exclusion criteria established, and that might limit creativity and innovation (Wang and Chugh, 2014; Nolan and Garavan, 2016). Second, in this review, four independent coders carefully clustered the findings of the analyzed studies, although other authors might have organized factors differently. Despite these limitations, this paper is a first attempt to provide a global picture of successful career transitions, and their antecedents, as well as the state of the art.

## Author contributions

AM: conceptualisation, methodology, formal analysis, and writing – original draft. SC: conceptualisation and writing – review & editing. ZG, CT, and DJ: investigation and data curation. All authors contributed to the article and approved the submitted version.

## Funding

This study was partly funded by the Institute of Adult Learning (IAL) Singapore (IAL Workforce Development Applied Research Fund grant number GA19-03).

## Conflict of interest

The authors declare that the research was conducted in the absence of any commercial or financial relationships that could be construed as a potential conflict of interest.

## Publisher’s note

All claims expressed in this article are solely those of the authors and do not necessarily represent those of their affiliated organizations, or those of the publisher, the editors and the reviewers. Any product that may be evaluated in this article, or claim that may be made by its manufacturer, is not guaranteed or endorsed by the publisher.
