# Supplementary material for: When is a career transition successful? a systematic literature review and outlook (1980–2022)
Source: Front Psychol. 2023 Sep 20;14:1141202. doi: 10.3389/fpsyg.2023.1141202 (PMC10552927; doi:10.3389/fpsyg.2023.1141202)
Supplement: Supplementary file 1 [file Data_Sheet_1.docx]

**References**

Armstrong-Stassen, M. (2003). Job transfer during organizational downsizing: A comparison of promotion and lateral transfers. *Group & Organization Management*, *28*(3), 392-415.

Arthur, M. B. (1994). The boundaryless career: A new perspective for organizational inquiry. *Journal of organizational behavior*, 295-306.

Ashforth, B. (2000). *Role transitions in organizational life: An identity-based perspective*. London: Routledge.

Bakker, A. B., Demerouti, E., De Boer, E., & Schaufeli, W. B. (2003). Job demands and job resources as predictors of absence duration and frequency. *Journal of vocational behavior*, *62*(2), 341-356.

Baruch, Y., Altman, Y., & Tung, R. L. (2016). Career mobility in a global era: Advances in managing expatriation and repatriation. *Academy of Management Annals*, *10*(1), 841-889.

Becker, G. S. (1962). Investment in human capital: A theoretical analysis. *Journal of political economy*, *70*(5, Part 2), 9-49.

Beehr, T.A. (2014). To retire or not to retire: That is not the question. *Journal of Organizational Behavior*, *35*(8), 1093-1108.

Bellah, R. N., Madsen, R., Sullivan, W. M., Swidler, A., & Tipton, S. M. (1996). *Habits of the heart: individualism and commitment in American life: updated edition with a new introduction*. Univ of California Press.

Bilgili, T.V., Calderon, C.J., Allen, D.G., & Kedia, B.L. (2017). Gone with the wind: A meta-analytic review of executive turnover, its antecedents, and post-acquisition performance. *Journal of Management*, *43*(6), 1966-1997.

Blustein, D.L., Chaves, A.P., Diemer, M.A., Gallagher, L.A., Marshall, K.G., Sirin, S., & Bhati, K.S. (2002). Voices of the forgotten half: The role of social class in the school-to-work transition. *Journal of Counseling Psychology*, *49*(3), 311-323.

Briscoe, J.P., Hall, D.T. & DeMuth, R.L.F. (2006). Protean and boundaryless careers: An empirical exploration. *Journal of Vocational Behavior*, *69*(1), 30-47.

Bynner, J.M. (1997). Basic skills in adolescents' occupational preparation. *The Career Development Quarterly*, *45*(4), 305-321.

Bynner, J., & Parsons, S. (2002). Social exclusion and the transition from school to work: The case of young people not in education, employment, or training (NEET). *Journal of Vocational Behavior*, *60*(2), 289-309.

Callister, R.R., Kramer, M.W., & Turban, D.B. (1999). Feedback seeking following career transitions. *Academy of Management Journal*, *42*(4), 429-438.

Campion, E.D., Campion, M.A., & Campion, M.C. (2021). A human capital‐based framework of career, well‐being, and social information reasons for managerial lateral job assignment preferences. *Human Resource Management*. Early view.

Cawsey, T., Deszca, G., & Mazerolle, M. (1995). The portfolio career is a response to a changing job market. *Journal of Career Planning and Employment, 56*(1), 41-46.

Coles, B. (2000). Changing patterns of youth transitions: Vulnerable groups, welfare careers and social. In Bynner, J., & Silbereisen, R. K. (Eds.) *Adversity and Challenge in Life in the New Germany and in England* (pp. 268-288). Basingstoke: Macmillan.

Chugh, A. (2021). What is “The Great Resignation”? An expert explains. The World Economic Forum. Available at: <https://www.weforum.org/agenda/2021/11/what-is-the-great-resignation-and-what-can-we-learn-from-it/> (accessed on February 1, 2022).

Crites, J. O. (1969). *Vocational psychology the study of vocational behavior and dev*. McGraw-Hill.

Crites, J. O. (1976). A comprehensive model of career development in early adulthood. *Journal of vocational behavior*, *9*(1), 105-118.

Deci, E. L., & Ryan, R. M. (2000). The" what" and" why" of goal pursuits: Human needs and the self-determination of behavior. Psychological inquiry, 11(4), 227-268.

De Vos, A., Akkermans, J., & Van Der Heijden, B.I.J.M. (2019). From occupational choice to career crafting. In Gunz, H., Lazarova, M., & Mayrhofer, W. (Eds.) *The Routledge companion to career studies* (pp.128-142). London: Routledge.

De Vos, A., Jacobs, S., & Verbruggen, M. (2021). Career transitions and employability. *Journal of Vocational Behavior*, *126*. Early view.

Eby, L.T., & Dematteo, J.S. (2000). When the type of move matters: Employee outcomes under various relocation situations. *Journal of Organizational Behavior, 21*(6), 677-687.

Ellis, A.M., Bauer, T.N., Mansfield, L.R., Erdogan, B., Truxillo, D.M. & Simon, L.S. (2015). Navigating uncharted waters: Newcomer socialization through the lens of stress theory. *Journal of Management*, *41*(1), 203-235.

Fang, R., Duffy, M.K., & Shaw, J.D. (2011). The organizational socialization process: Review and development of a social capital model. *Journal of Management*, *37*(1), 127-152.

Feather, N.T., & O'Brien, G.E. (1986). A longitudinal study of the effects of employment and unemployment on school‐leavers. *Journal of Occupational Psychology*, *59*(2), 121-144.

Feintuch, A. (1955). Improving the employability and attitudes of" difficult-to-place" persons. *Psychological Monographs: General and Applied*, *69*(7), 1.

Festinger, L. (1954). A theory of social comparison processes. *Human relations*, *7*(2), 117-140.

Gesthuizen, M., & Dagevos, J. (2008). Mismatching of persons and jobs in the Netherlands: consequences for the returns to mobility. *Work, Employment and Society*, *22*(3), 485-506.

Gowan, M.A. (2014). Moving from job loss to career management: The past, present, and future of involuntary job loss research. *Human Resource Management Review*, *24*(3), 258-270.

Heppner, M. J. (1998). The career transitions inventory: Measuring internal resources in adulthood. *Journal of Career Assessment*, *6*(2), 135-145.

Heppner, M. J., & Jung, A. K. (2013). Gender and social class: Powerful predictors of a life journey. In *Handbook of vocational psychology* (pp. 81-102). Routledge.

Heslin, P. A. (2005). Conceptualizing and evaluating career success. *Journal of Organizational Behavior: The International Journal of Industrial, Occupational and Organizational Psychology and Behavior*, *26*(2), 113-136.

Hirschi, A. (2010). The role of chance events in the school-to-work transition: The influence of demographic, personality and career development variables. *Journal of Vocational Behavior*, *77*(1), 39-49.

Hite, L.M., & McDonald, K. (2020). Careers after COVID-19: challenges and changes. *Human Resource Development International, 23*(4), 427-437.

Hom, P.W., Lee, T.W., Shaw, J.D., & Hausknecht, J.P. (2017). One hundred years of employee turnover theory and research. *Journal of Applied Psychology*, *102*(3), 530-545.

Kalleberg, A.L., & Mastekaasa, A. (2001). Satisfied movers, committed stayers: The impact of job mobility on work attitudes in Norway. *Work and Occupations*, *28*(2), 183-209.

Klotz, A.C. (2021). The Covid vaccine means a return to work. And a wave of resignations.
NBC News THINK. Available at: https://www.nbcnews.com/think/opinion/covid-vaccine-means-return-work-
wave-resignations-ncna1269018 (accessed on February 1, 2022).

Kraimer, M., Bolino, M., & Mead, B. (2016). Themes in expatriate and repatriate research over four decades: what do we know and what do we still need to learn?. *Annual Review of Organizational Psychology and Organizational Behavior*, *3*, 83-109.

Kristof, A. L. (1996). Person‐organization fit: An integrative review of its conceptualizations, measurement, and implications. *Personnel psychology*, *49*(1), 1-49.

Latack, J.C. (1984). Career transitions within organizations: An exploratory study of work, nonwork, and coping strategies. *Organizational Behavior and Human Performance*, *34*(3), 296-322.

Lent, R.W., Brown, S.D., & Hackett, G. (1994). Toward a unifying social cognitive theory of career and academic interest, choice, and performance. *Journal of Vocational Behavior*, *45*(1), 79-122.

Lent, R.W., Lopez Jr, A.M., Lopez, F.G. & Sheu, H.B. (2008). Social cognitive career theory and the prediction of interests and choice goals in the computing disciplines. *Journal of Vocational Behavior*, *73*(1), 52-62.

Ling, T.J., & O'Brien, K.M. (2013). Connecting the forgotten half: The school-to-work transition of noncollege-bound youth. *Journal of Career Development*, *40*(4), 347-367.

Locke, E. A., & Latham, G. P. (1990). *A theory of goal setting & task performance*. Prentice-Hall, Inc.

Louis, M.R. (1980). Career transitions: Varieties and commonalities. *Academy of Management Review*, *5*(3), 329-340.

Lyons, S. T., Schweitzer, L., & Ng, E. S. (2015). How have careers changed? An investigation of changing career patterns across four generations. *Journal of Managerial Psychology, 30*(1), 8-21.

Moher, D., Liberati, A., Tetzlaff, J., & Altman, D.G. (2009). Preferred reporting items for systematic reviews and meta-analyses: the PRISMA statement. *PLoS Med*, *6*(7).

Monear, D. (2020). Strategies for a Changing Labor Market: Reskilling and Upskilling for Career Advancement in the Post-COVID-19 Economy. Higher Education and the Labor Market. Washington Student Achievement Council.

Moyle, P., & Parkes, K. (1999). The effects of transition stress: A relocation study. *Journal of Organizational Behavior*, *20*(5), 625-646.

Ngai, E.W.T., Moon, K.K., Riggins, F.J., & Candace, Y.Y. (2008). RFID research: An academic literature review (1995–2005) and future research directions. *International Journal of Production Economics*, *112*(2), 510-520.

Nicholson, N., & West, M. (1988). *Managerial job change: Men and women in transition*. Cambridge: Cambridge University Press.

Nicholson, N. (1984). A theory of work role transitions. *Administrative Science Quarterly*, 172-191.

Nolan, C.T., & Garavan, T.N. (2016). Human resource development in SMEs: a systematic review of the literature. *International Journal of Management Reviews*, *18*(1), 85-107.

O'Neil, J.M., Fishman, D.M., & Kinsella-Shaw, M. (1987). Dual-career couples' career transitions and normative dilemmas: A preliminary assessment model. *The Counseling Psychologist*, *15*(1), 50-96.

Perera, H. N., & McIlveen, P. (2014). The role of optimism and engagement coping in college adaptation: A career construction model. *Journal of Vocational Behavior*, *84*(3), 395-404.

Pinder, C.C. and Schroeder, K.G. (1987). Time to proficiency following job transfers. *Academy of Management Journal*, *30*(2), 336-353.

Reio Jr, T. G., & Wiswell, A. (2000). Field investigation of the relationship among adult curiosity, workplace learning, and job performance. *Human Resource Development Quarterly, 11*(1), 5-30.

Ranta, M., Chow, A., & Salmela-Aro, K. (2013). Trajectories of life satisfaction and the financial situation in the transition to adulthood. *Longitudinal and Life Course Studies*, *4*(1), 57-77.

Saks, A.M., & Ashforth, B.E. (1999). Effects of individual differences and job search behaviors on the employment status of recent university graduates. *Journal of Vocational Behavior*, *54*(2), 335-349.

Saks, A.M. (1995). Longitudinal field investigation of the moderating and mediating effects of self-efficacy on the relationship between training and newcomer adjustment. *Journal of Applied Psychology*, *80*(2), 211-225.

Sarason, S.B. (1977). *Work, aging, and social change: Professionals and the one life-one career imperative*. New York: Free Press.

Savickas, M.L. (1994). Measuring career development: Current status and future directions. *The Career Development Quarterly*, *43*(1), pp.54-62.

Savickas, M.L. (1999). The transition from school to work: A developmental perspective. *The Career Development Quarterly*, *47*(4), 326-336.

Savickas, M.L. (2005). The theory and practice of career construction. In Brown, S.D., & Lent, R.W. (Eds.) *Career development and counseling: Putting theory and research to work* (pp. 147-186). New York: Wiley.

Schlossberg, N.K. (1981). A model for analyzing human adaptation to transition. *The Counseling Psychologist*, *9*(2), 2-18.

Schlossberg, N.K. (2011). The challenge of change: The transition model and its applications. *Journal of Employment Counseling*, *48*(4), 159-162.

Settersten, Jr, R.A. (2005). Toward a stronger partnership between life-course sociology and life-span psychology. *Research in Human Development*, *2*(1-2), 25-41.

Shirani, A. (2019). Upskilling and retraining in data analytics: A skill-adjacency analysis for career paths. Issues in Information Systems, 20(4).

Sullivan, S.E., & Al Ariss, A. (2021). Making sense of different perspectives on career transitions: A review and agenda for future research. *Human Resource Management Review*, *31*(1). Early view.

Super, D. E. (1957). The psychology of careers; an introduction to vocational development.

Takeuchi, R. (2010). A critical review of expatriate adjustment research through a multiple stakeholder views: Progress, emerging trends, and prospects. *Journal of Management*, *36*(4), 1040-1064.

Upadyaya, K., & Salmela-Aro, K. (2017). Developmental dynamics between young adults’ life satisfaction and engagement with studies and work. *Longitudinal and Life Course Studies*, *8*(1), 20-34.

Van Dierendonck, D., & Jacobs, G. (2012). Survivors and victims, a meta‐analytical review of fairness and organizational commitment after downsizing. *British Journal of Management*, *23*(1), 96-109.

Verbruggen, M., De Cooman, R., & Vansteenkiste, S. (2015). When and why are internal job transitions successful? Transition challenges, hindrances, and resources influencing motivation and retention through basic needs satisfaction. *Group & Organization Management*, *40*(6), 744-775.

Vuolo, M., Staff, J., & Mortimer, J.T. (2012). Weathering the great recession: Psychological and behavioral trajectories in the transition from school to work. *Developmental Psychology*, *48*(6), 1759-1773.

Wanberg, C.R., & Kammeyer-Mueller, J. (2008). A self-regulatory perspective on navigating career transitions. In Kanfer, R., Chen., G., & Pritchard, R.D. (Eds.) Work motivation: Past, present and future (pp. 435-460). New York: Routledge.

Wang, C.L., & Chugh, H. (2014). Entrepreneurial learning: Past research and future challenges. *International Journal of Management Reviews*, *16*(1), 24-61.

Wang, M., & Shi, J. (2014). Psychological research on retirement. *Annual Review of Psychology*, *65*, 209-233.

West, M.A., Nicholson, N. & Rees, A. (1987). Transitions into newly created jobs. *Journal of Occupational Psychology*, *60*(2), 97-113.

Wrzesniewski, A., LoBuglio, N., Dutton, J. E., & Berg, J. M. (2013). Job crafting and cultivating positive meaning and identity in work. *Advances in positive organizational psychology, 1,* 281–302.

**Appendix B**

*Articles included in the systematic literature review*

[1] Abkhezr, P., McMahon, M., & Rossouw, P. (2015). Youth with refugee backgrounds in Australia: Contextual and practical considerations for career counsellors. *Australian Journal of Career Development, 24*(2), 71-80. doi:10.1177/1038416215584406

[2] Ahn, J., Dik, B. J., & Hornback, R. (2017). The experience of career change driven by a sense of calling: An Interpretative Phenomenological Analysis approach. *Journal of Vocational Behavior, 102*, 48-62. doi:10.1016/j.jvb.2017.07.003

[3] Akos, P., Hutson, B., & Leonard, A. J. (2022). The Relationship Between Work Study and Career Development for Undergraduate Students. *Journal of Career Development*, *49*(5), 1097–1107. <https://doi.org/10.1177/08948453211012787>

[4] Alfano V., G. G., Pinto M. (2021). Non-academic employment and matching satisfaction among PhD graduates with high intersectoral mobility potential. *International Journal of Manpower, 42*(7), 1202-1223.

[5] Alon, S., & Haberfeld, Y. (2007). Labor force attachment and the evolving wage gap between white, black, and Hispanic young women. *Work and Occupations, 34*(4), 369-398.

[5] Arar, K. (2017). How novice principals face the challenges of principalship in the Arab education system in Israel. *Journal of Career Development, 45*(6), 580-596. doi:10.1177/0894845317726392

[6] Baer, R. M., Daviso, A. W., Flexer, R. W., McMahan Queen, R., & Meindl, R. S. (2011). Students with intellectual disabilities. *Career Development for Exceptional Individuals, 34*(3), 132-141. doi:10.1177/0885728811399090

[7] Ballo, J. G. (2020). Labour market participation for young people with disabilities: The impact of gender and higher education. *Work, Employment and Society,* *34*(2), 336-355. doi:10.1177/0950017019868139

[8] Barclay, S. R., & Stoltz, K. B. (2016). The life-design group: a case study assessment. *The Career Development Quarterly, 64*(1), 83-96. doi:10.1002/cdq.12043

[9] Baruch, Y., & Campbell Quick, J. (2009). Setting sail in a new direction. Career transitions of US Navy Admirals to the civilian sector. *Personnel Review, 38*(3), 270-285. doi:10.1108/00483480910943331

[10] Becker, K., Bish, A., Abell, D., McCormack, M., & Smidt, M. (2022). Supporting Australian veteran transition: Career construction through a person-environment fit perspective. *The International Journal of Human Resource Management*, 1–25. <https://doi.org/10.1080/09585192.2022.2077127>

[11] Belle, M. A., Antwi, C. O., Ntim, S. Y., Affum-Osei, E., & Ren, J. (2021). Am I gonna get a job? graduating students’ psychological capital, coping styles, and employment Anxiety. *Journal of Career Development*. doi:10.1177/08948453211020124

[12] Betz, N. E., & Wolfe, J. B. (2016). Measuring confidence for basic domains of vocational activity in high school students. *Journal of Career Assessment, 13*(3), 251-270. doi:10.1177/1069072705274951

[13] Bimrose, J., Brown, A., Mulvey, R., Kieslinger, B., Fabian, C. M., Schaefer, T., . . . Dewanti, R. T. (2019). Transforming identities and co-constructing careers of career counselors. *Journal of Vocational Behavior, 111*, 7-23. doi:10.1016/j.jvb.2018.07.008

[14] Black, K., & Warhurst, R. (2018). Career transition as identity learning: an autoethnographic understanding of human resource development. *Human Resource Development International, 22*(1), 25-43. doi:10.1080/13678868.2018.1444005

[15] Boveda, I., & Metz, A. J. (2016). Predicting end-of-career transitions for baby boomers nearing retirement age. *The Career Development Quarterly, 64*(2), 153-168. doi:10.1002/cdq.12048

[16] Bown‐Wilson, D., Loretto, W., & Parry, E. (2013). Career progression in older managers. *Employee Relations, 35*(3), 309-321. doi:10.1108/01425451311320495

[17] Bradley, L. J. (1985). Making the transition from prison to community: Importance of career exploration. *Journal of Employment Counseling, , 22*(3), 110-116.

[18] Briggs, A. Q. (2019). “We had support from our brothers”: a critical race counter-narrative inquiry into second-generation Black Caribbean male youth responses to discriminatory work pathways. *Journal of Education and Work, 32*(4), 377-392. doi:10.1080/13639080.2019.1624696

[19] Brown, C., Hooley, T., & Wond, T. (2020). Building career capital: developing business leaders' career mobility. *Career Development International, 25*(5), 445-459. doi:10.1108/cdi-07-2019-0186

[20] Bruce, R. A., & Scott, S. G. (1994). Varieties and commonalities of career transitions: Louis′ typology revisited. *Journal of Vocational Behavior, 45*(1), 17-40.

[21] Buyukgoze-Kavas, A. (2014). Validation of the Career Adapt-Abilities Scale-Turkish form and its relation to hope and optimism. *Australian Journal of Career Development, 23*(3), 125-132. doi:10.1177/1038416214531931

[22] Cabrera, E. F. (2007). Opting out and opting in: understanding the complexities of women's career transitions. *Career Development International, 12*(3), 218-237. doi:10.1108/13620430710745872

[23] Cahill, K. E., Giandrea, M. D., & Quinn, J. F. (2017). To what extent is gradual retirement a product of financial necessity? *Work, Aging and Retirement, 3*(1), 25-54. doi:10.1093/workar/waw027

[24] Cai, D., Li, Z., Xu, L., Fan, L., Wen, S., Li, F., Guan, Z., & Guan, Y. (2023). Sustaining newcomers’ career adaptability: The roles of socialization tactics, job embeddedness and career variety. *Journal of Occupational and Organizational Psychology*, joop.12423. <https://doi.org/10.1111/joop.12423>

[25] Cappellini, E., Maitino, M., Patacchini, V., & Sciclone, N. (2019). Are traineeships stepping-stones for youth working careers in Italy? *International Journal of Manpower, 40*(8), 1389-1410. doi:10.1108/ijm-03-2018-0099

[26] Carvalho, L., Mourão, L., & Freitas, C. (2023). Career counseling for college students: Assessment of an online and group intervention. *Journal of Vocational Behavior*, *140*, 103820. <https://doi.org/10.1016/j.jvb.2022.103820>

[27] Chamandy, M., & Gaudreau, P. (2019). Career doubt in a dual-domain model of coping and progress for academic and career goals. *Journal of Vocational Behavior, 110*, 155-167. doi:10.1016/j.jvb.2018.11.008

[28] Chan, C.-C. (2018). Factors affecting career goals of Taiwanese college athletes from perspective of social cognitive career theory. *Journal of Career Development, 47*(2), 193-206. doi:10.1177/0894845318793234

[29] Chudzikowski, K. (2012). Career transitions and career success in the 'new' career era. *Journal of Vocational Behavior, 81*(2), 298-306. doi:10.1016/j.jvb.2011.10.005

[30] Clarke, M. (2009). Plodders, pragmatists, visionaries and opportunists: career patterns and employability. *Career Development International, 14*(1), 8-28. doi:10.1108/13620430910933556

[31] Clemens, E. V., & Milsom, A. S. (2008). Enlisted service members' transition into the civilian world of work: A cognitive information processing approach. *The Career Development Quarterly, 56*(3), 246-256.

[32] Cohen, L., & Mallon, M. (1999). The transition from organisational employment to portfolio working: Perceptions of ‘boundarylessness’. *Work, employment and society, 13*(2), 329-352.

[33] Comi, S. L., Grasseni, M., & Origo, F. (2022). Sometimes it works: The effect of a reform of the short vocational track on school-to-work transition. *International Journal of Manpower*, *43*(7), 1601–1619. <https://doi.org/10.1108/IJM-06-2021-0391>

[34] Cordeiro, P. M., Paixão, M. P., Lens, W., Lacante, M., & Luyckx, K. (2015). Cognitive–motivational antecedents of career decision-making processes in Portuguese high school students: A longitudinal study. *Journal of Vocational Behavior, 90*, 145-153. doi:10.1016/j.jvb.2015.08.005

[35] Cordeiro, P. M. G., Paixão, M. P., Lens, W., Lacante, M., & Luyckx, K. (2016). Parenting styles, identity development, and adjustment in career transitions. *Journal of Career Development, 45*(1), 83-97. doi:10.1177/0894845316672742

[36] Corrales-Herrero, H., & Rodríguez-Prado, B. (2014). The role of part-time employment among young people with a non-university education in Spain. *Journal of Education and Work, 29*(2), 167-198. doi:10.1080/13639080.2014.918943

[37] Coutinho, M. J., Oswald, D. P., & Best, A. M. . (2006 ). Differences in outcomes for female and male students in special education. *Career Development for Exceptional Individuals, 29*(1 ), 48-59.

[38] Creed, P. A., & Blume, K. (2012). Compromise, Well-Being, and Action Behaviors in Young Adults in Career Transition. *Journal of Career Assessment, 21*(1), 3-19. doi:10.1177/1069072712453830

[39] Cummins, P., & O’Boyle, I. (2014). Psychosocial factors involved in transitions from college to postcollege careers for male NCAA Division-1 basketball players. *Journal of Career Development, 42*(1), 33-47. doi:10.1177/0894845314532713

[40] Davies, E., Loretto, W., & Jenkins, A. (2013). The work‐to‐retirement transition of academic staff: attitudes and experiences. *Employee Relations, 35*(3), 322-338. doi:10.1108/01425451311320503

[41] Day, J. C. (2015). Transitions to the top. *Work and Occupations, 42*(4), 408-446. doi:10.1177/0730888415580651

[42] Dekker, R., de Grip, A., & Heijke, H. (2002). The effects of training and overeducation on career mobility in a segmented labour market. *International Journal of Manpower, 23*(2), 106-125. doi:10.1108/01437720210428379

[43] Dick, P. (2010). The transition to motherhood and part-time working: mutuality and incongruence in the psychological contracts existing between managers and employees. *Work, Employment and Society, 24*(3), 508-525. doi:10.1177/0950017010371648

[44] Dietrich, J., Jokisaari, M., & Nurmi, J.-E. (2012). Work-related goal appraisals and stress during the transition from education to work. *Journal of Vocational Behavior, 80*(1), 82-92. doi:10.1016/j.jvb.2011.07.004

[45] Dingemans, E., & Henkens, K. (2014). Involuntary retirement, bridge employment, and satisfaction with life: A longitudinal investigation. *Journal of Organizational Behavior, 35*(4), 575-591. doi:10.1002/job.1914

[46] Dipeolu, A. O. (2007). Career instruments and high school students with learning disabilities: Support for the utility of three vocational measures. *Journal of Career Development, 34*(1), 59-78.

[47] Dipeolu, A. O., Hargrave, S., Sniatecki, J. L., & Donaldson, J. (2012). Improving prediction of significant career‐related constructs for high school students with learning disabilities. *The Career Development Quarterly, 60*(3), 207-220.

[48] Donald, W. E., Ashleigh, M. J., & Baruch, Y. (2018). Students’ perceptions of education and employability. *Career Development International, 23*(5), 513-540. doi:10.1108/cdi-09-2017-0171

[49] Drishti, E., & Carmichael, F. (2023). Dead-end jobs or steppingstones? Precarious work in Albania. *Personnel Review*, *52*(1), 99–120. <https://doi.org/10.1108/PR-04-2021-0275>

[50] Duberley, J., & Carmichael, F. (2016). Career pathways into retirement in the UK: Linking older women's pasts to the present. *Gender, Work & Organization, 23*(6), 582-599. doi:10.1111/gwao.12144

[51] Duberley, J., Mallon, M., & Cohen, L. (2006). Exploring career transitions: accounting for structure and agency. *Personnel Review, 35*(3), 281-296. doi:10.1108/00483480610656694

[52] Durbin, S., & Tomlinson, J. (2011). Female part-time managers: networks and career mobility. *Work, Employment and Society, 24*(4), 621-640. doi:10.1177/0950017010380631

[53] Durbin, S., & Tomlinson, J. (2014). Female part-time managers: careers, mentors and role models. *Gender, Work & Organization, 21*(4), 308-320. doi:10.1111/gwao.12038

[54] Elchardus, M., & Smits, W. (2008). The vanishing flexible: ambition, self-realization and flexibility in the career perspectives of young Belgian adults. *Work, Employment and Society, 22*(2), 243-262. doi:10.1177/0950017008089103

[55] Evertsson, M., Grunow, D., & Aisenbrey, S. (2015). Work interruptions and young women’s career prospects in Germany, Sweden and the US. *Work, Employment and Society, 30*(2), 291-308. doi:10.1177/0950017015598283

[56] Farčnik, D., Parodi, G., & Domadenik, P. (2012). Has the Bologna reform enhanced the employability of graduates? Early evidence from Slovenia. *International Journal of Manpower, 33*(1), 51-75. doi:10.1108/01437721211212529

[57] Farivar, F., Coffey, J., & Cameron, R. (2019). International graduates and the change of initial career mobility intentions. *Personnel Review, 48*(4), 1061-1078. doi:10.1108/pr-01-2017-0007

[58] Fasbender, U., Wang, M., Voltmer, J.-B., & Deller, J. (2015). The meaning of work for post-retirement employment decisions. *Work, Aging and Retirement*. doi:10.1093/workar/wav015

[59] Fehring, H., & Herring, K. (2013). TheWorking Livesproject: a window into Australian education and workforce participation. *Journal of Education and Work, 26*(5), 494-513. doi:10.1080/13639080.2012.693585

[60] Feij, J. A., Whitely, W. T., Peiró, J. M., & Taris, T. W. . (1995). The development of career-enhancing strategies and content innovation: A longitudinal study of new workers. . *Journal of Vocational Behavior, 46*(3 ), 231-256.

[61] Fiaschi, D., & Tealdi, C. (2022). Young people between education and the labour market during the COVID-19 pandemic in Italy. *International Journal of Manpower*, *43*(7), 1719–1757. <https://doi.org/10.1108/IJM-06-2021-0352>

[62] Flannery, K. B., Yovanoff, P., Benz, M. R., & Kato, M. M. (2008). Improving employment outcomes of individuals with disabilities through short-term postsecondary training. *Career Development for Exceptional Individuals, 31*(1), 26-36.

[63] Flexer, R. W., Daviso, A. W., Baer, R. M., McMahan Queen, R., & Meindl, R. S. (2011). An epidemiological model of transition and postschool outcomes. *Career Development for Exceptional Individuals, 34*(2), 83-94. doi:10.1177/0885728810387922

[64] Forret, M. L., Sullivan, S. E., & Mainiero, L. A. (2010). Gender role differences in reactions to unemployment: Exploring psychological mobility and boundaryless careers. *Journal of Organizational Behavior, 31*(5), 647-666. doi:10.1002/job.703

[65] Fu, C., Cai, Y., Yang, Q., Pan, G., Xu, D., & Shi, W. (2022). Career Adaptability Development in the School-To-Work Transition. *Journal of Career Assessment*, 106907272211203. <https://doi.org/10.1177/10690727221120366>

[66] Furunes, T., Mykletun, R. J., Solem, P. E., de Lange, A. H., Syse, A., Schaufeli, W. B., & Ilmarinen, J. (2015). Late career decision-making: A qualitative panel study. *Work, Aging and Retirement, 1*(3), 284-295. doi:10.1093/workar/wav011

[67] Galais, N., & Moser, K. (2018). Temporary agency workers stepping into a permanent position: social skills matter. *Employee Relations, 40*(1), 124-138. doi:10.1108/er-08-2016-0158

[68] Gamboa, V., Paixão, M. P., da Silva, J. T., & Taveira, M. d. C. (2020). Career goals and internship quality among VET students. *Journal of Career Development, 48*(6), 910-925. doi:10.1177/0894845320902269

[69] Gati, I., Ryzhik, T., & Vertsberger, D. (2013). Preparing young veterans for civilian life: The effects of a workshop on career decision-making difficulties and self-efficacy. *Journal of Vocational Behavior, 83*(3), 373-385. doi:10.1016/j.jvb.2013.06.001

[70] Ghosh, A., & Fouad, N. A. (2017). Career adaptability and social support among graduating college seniors. *The Career Development Quarterly, 65*(3), 278-283.

[71] Ghosh, A., Kessler, M., Heyrman, K., Opelt, B., Carbonelli, M., & Fouad, N. A. (2019). Student veteran career transition readiness, career adaptability, and academic and life satisfaction. *The Career Development Quarterly, 67*(4), 365-371. doi:10.1002/cdq.12205

[72] Gilkison, B., & Drummond, R. J. (1988). Academic self‐concept of older adults in career transition. *Journal of Employment Counseling, , 25*(1), 24-29.

[73] Gold, M., & Fraser, J. (2002 ). Managing self-management: Successful transitions to portfolio careers. *Work, employment and society., 16*(4 ), 579-597.

[74] Green, Z. A., Noor, U., & Hashemi, M. N. (2019). Furthering proactivity and career adaptability among university students: Test of intervention. *Journal of Career Assessment, 28*(3), 402-424. doi:10.1177/1069072719870739

[75] Guan, Y., Zhou, X., Zheng, Y., Wen, S., Fu, Y., Hu, N., Fu, A., Han, Y., & Wang, Z. (2022). Role of perceived events in university graduates’ job search self-efficacy and success. *Journal of Vocational Behavior*, *136*, 103741. <https://doi.org/10.1016/j.jvb.2022.103741>

[76] Han, H., & Rojewski, J. W. (2014). Gender-specific models of work-bound Korean adolescents’ social supports and career adaptability on subsequent job satisfaction. *Journal of Career Development, 42*(2), 149-164. doi:10.1177/0894845314545786

[77] Heinz, W. R. (2002). Transition discontinuities and the biographical shaping of early work careers. *Journal of Vocational Behavior, 60*(2), 220-240. doi:10.1006/jvbe.2001.1865

[78] Hennekam, S. (2017). Dealing with multiple incompatible work-related identities: the case of artists. *Personnel Review, 46*(5), 970-987. doi:10.1108/pr-02-2016-0025

[79] Heppner, M. J., Fuller, B. E., & Multon, K. D. (1998). Adults in involuntary career transition: An analysis of the relationship between the psychological and career domains. *Journal of Career Assessment, 6*(3), 329-346.

[80] Heppner, M. J., Lee, D.-g., Paul Heppner, P., McKinnon, L. C., Multon, K. D., & Gysbers, N. C. (2004). The role of problem-solving appraisal in the process and outcome of career counseling. *Journal of Vocational Behavior, 65*(2), 217-238. doi:10.1016/s0001-8791(03)00100-3

[81] Hirschi, A. (2010). The role of chance events in the school-to-work transition: The influence of demographic, personality and career development variables. *Journal of Vocational Behavior, 77*(1), 39-49. doi:10.1016/j.jvb.2010.02.002

[82] Hirschi, A., & Vondracek, F. W. (2009). Adaptation of career goals to self and opportunities in early adolescence. *Journal of Vocational Behavior, 75*(2), 120-128. doi:10.1016/j.jvb.2009.05.005

[83] Hlad′o, P., Kvasková, L., Ježek, S., Hirschi, A., & Macek, P. (2019). Career adaptability and social support of vocational students leaving upper secondary school. *Journal of Career Assessment, 28*(3), 478-495. doi:10.1177/1069072719884299

[84] Howes, L. M., & Goodman-Delahunty, J. (2013). Life Course Research Design: Exploring Career Change Experiences of Former School Teachers and Police Officers. *Journal of Career Development, 41*(1), 62-84. doi:10.1177/0894845312474370

[85] Hui, T., Yuen, M., & Chen, G. (2018a). Career‐Related filial piety and career adaptability in hong kong university students. The Career Development Quarterly, 66(4), 358-370. doi:10.1002/cdq.12156

[86] Hui, T., Yuen, M., & Chen, G. (2018b). Career Adaptability, Self-Esteem, and Social Support Among Hong Kong University Students. *The Career Development Quarterly, 66*(2), 94-106. doi:10.1002/cdq.12118

[87] Hur, H., Maurer, J. A., & Hawley, J. (2019). The role of education, occupational match on job satisfaction in the behavioral and social science workforce. *Human Resource Development Quarterly, 30*(3), 407-435. doi:10.1002/hrdq.21343

[88] Hurst, J. L., & Good, L. K. (2009). Generation Y and career choice. *Career Development International, 14*(6), 570-593. doi:10.1108/13620430910997303

[89] Hwang, M. H., Lee, J., & Jung, A.-K. (2018). Career development of lower social class adolescents in South Korea. *Journal of Career Development, 46*(5), 516-530. doi:10.1177/0894845318780361

[90] Ishikawa, Y., Mizuno, M., & Amundson, N. E. (2009). Career awareness of new graduates and recruiting activities by companies in Japan. *Journal of Employment Counseling, 46*(2), 62-72.

[91] Jackson, D. (2014). Modelling graduate skill transfer from university to the workplace. *Journal of Education and Work, 29*(2), 199-231. doi:10.1080/13639080.2014.907486

Jackson, D., & Li, I. (2022). Transition to work, mismatch and underemployment among graduates: An Australian longitudinal study. *International Journal of Manpower*, *43*(7), 1516–1539. <https://doi.org/10.1108/IJM-03-2021-0195>

[92] Johnson, A. M., & Jackson, P. R. (2012). Golden parachutes: Changing the experience of unemployment for managers. *Journal of Vocational Behavior, 80*(2), 474-485. doi:10.1016/j.jvb.2011.08.001

[93] Jose Cabral‐Cardoso, C. (2001). Too academic to get a proper job? The difficult transition of PhDs to the “real world” of industry. *Career Development International, 6*(4), 212-217. doi:10.1108/13620430110397505

[94] Karpefors, M., & van Riemsdijk, M. (2019). ‘We are not free here…’ – Palestinian IT students’ (im)mobile transition from university to employment or further education. *Journal of Education and Work, 33*(1), 19-32. doi:10.1080/13639080.2019.1704705

[95] Karpur, A., Brewer, D., & Golden, T. (2013). Critical program elements in transition to adulthood. *Career Development and Transition for Exceptional Individuals, 37*(2), 119-130. doi:10.1177/2165143413476880

[96] Kashefpakdel, E. T., & Percy, C. (2016). Career education that works: an economic analysis using the British Cohort Study. *Journal of Education and Work, 30*(3), 217-234. doi:10.1080/13639080.2016.1177636

[97] Kattenbach, R., Schneidhofer, T. M., Lucke, J., Latzke, M., Loacker, B., Schramm, F., & Mayrhofer, W. (2014). A quarter of a century of job transitions in Germany. *Journal of Vocational Behavior, 84*(1), 49-58. doi:10.1016/j.jvb.2013.11.001

[98] Kepir Sávoly, D. D., & Tuzgol Dost, M. (2020). Effectiveness of a school-to-work transition skills program in a collectivist culture. *Australian Journal of Career Development, 29*(2), 127-136. doi:10.1177/1038416220919882

[99] Kidd, J. M. (2008). Exploring the components of career well-being and the emotions associated with significant career experiences. *Journal of Career Development, 35*(2), 166-186.

[100] Kim, B., Kim, S. R., Yang, N. Y., Yaung, H., Ha, G. Y., Yang, J. Y., . . . Lee, S. M. (2016). Longitudinal relationships between planned happenstance skills and life adjustment and the moderating role of career barriers. *Journal of Career Development, 45*(3), 215-226. doi:10.1177/0894845316681078

[101] Kim, B., Lee, B. H., Ha, G., Lee, H. K., & Lee, S. M. (2015). Examining longitudinal relationships between dysfunctional career thoughts and career decision-making self-efficacy in school-to-work transition. *Journal of Career Development, 42*(6), 511-523. doi:10.1177/0894845315578903

[102] Kim, B., Rhee, E., Ha, G., Yang, J., & Lee, S. M. (2016). Tolerance of uncertainty: Links to happenstance, career decision self-efficacy, and career satisfaction. *The Career Development Quarterly, 64*(2), 140-152. doi:10.1002/cdq.12047

[103] Kim, M. H., & Beier, M. E. (2020). The college-to-career transition in STEM: An eleven-year longitudinal study of perceived and objective vocational interest fit. *Journal of Vocational Behavior, 123*. doi:10.1016/j.jvb.2020.103506

[104] Klug, K., Drobnič, S., & Brockmann, H. (2019). Trajectories of insecurity: Young adults' employment entry, health and well-being. *Journal of Vocational Behavior, 115*. doi:10.1016/j.jvb.2019.05.005

[105] Koen, J., Klehe, U.-C., & Van Vianen, A. E. M. (2012). Training career adaptability to facilitate a successful school-to-work transition. *Journal of Vocational Behavior, 81*(3), 395-408. doi:10.1016/j.jvb.2012.10.003

[106] Koen, J., Van Vianen, A., Klehe, U.-C., & Zikic, J. (2016). “A whole new future” – identity construction among disadvantaged young adults. *Career Development International, 21*(7), 658-681. doi:10.1108/cdi-02-2016-0019

[107] Kormanik, M. B. (2008). The stalled career: Addressing an organizational undiscussable. *Advances in Developing Human Resources, 10*(1), 50-69. doi:10.1177/1523422307310112

[108] Korte, R., Brunhaver, S., & Sheppard, S. (2015). (Mis)Interpretations of organizational socialization: the expectations and experiences of newcomers and managers. *Human Resource Development Quarterly, 26*(2), 185-208. doi:10.1002/hrdq.21206

[109] Kuron, L. K. J., Lyons, S. T., Schweitzer, L., & Ng, E. S. W. (2015). Millennials’ work values: differences across the school to work transition. *Personnel Review, 44*(6), 991-1009. doi:10.1108/pr-01-2014-0024

[110] Kvasková, L., Hlado, P., Palíšek, P., Šašinka, V., Hirschi, A., Ježek, S., & Macek, P. (2023). A Longitudinal Study of Relationships Between Vocational Graduates’ Career Adaptability, Career Decision-Making Self-Efficacy, Vocational Identity Clarity, and Life Satisfaction. *Journal of Career Assessment*, *31*(1), 27–49. <https://doi.org/10.1177/10690727221084106>

[111] Lahlouh, K., Lacaze, D., & Huaman-Ramirez, R. (2019). Bridge employment and full retirement intentions: the role of person-environment fit. *Personnel Review, 48*(6), 1469-1490. doi:10.1108/pr-02-2018-0067

[112] Lam, B. O.-Y., & Tang, H.-h. H. (2020). Making sense of ‘graduate employability’ in Hong Kong:A contextualized analysis of experience and interpretations of graduates of self-financing higher education institutions. *Journal of Education and Work, 34*(1), 14-28. doi:10.1080/13639080.2020.1858229

[113] Lane, A., & Lee, D. L. (2018). Career Transitions of highly skilled immigrants: two case studies. *The Career Development Quarterly, 66*(4), 315-328. doi:10.1002/cdq.12153

[114] LaPointe, K. (2013). Heroic career changers? Gendered identity work in career transitions. *Gender, Work & Organization, 20*(2), 133-146. doi:10.1111/j.1468-0432.2012.00601.x

[115] Lavallee, D. (2006). Career awareness, career planning, and career transition needs among sports coaches.. *Journal of Career Development, 33*(1), 66-79.

[116] Lehmann, W. (2019). Forms of capital in working-class students’ transition from university to employment. *Journal of Education and Work, 32*(4), 347-359. doi:10.1080/13639080.2019.1617841

[117] Lewis, K. V., Harris, C., Morrison, R., & Ho, M. (2015). The entrepreneurship-motherhood nexus: A longitudinal investigation from a boundaryless career perspective. *Career Development International, 20*(1), 21-37. doi:10.1108/cdi-07-2014-0090

[119] Lindstrom, L., Doren, B., Post, C., & Lombardi, A. (2013). Building career PATHS (Postschool Achievement Through Higher Skills) for Young women with disabilities. *The Career Development Quarterly, 61*(4), 330-338. doi:10.1002/j.2161-0045.2013.00059.x

[120] Lindstrom, L., Harwick, R. M., Poppen, M., & Doren, B. (2012). Gender gaps. *Career Development and Transition for Exceptional Individuals, 35*(2), 108-117. doi:10.1177/0885728812437737

[121] Lindstrom, L., Hirano, K. A., McCarthy, C., & Alverson, C. Y. (2014). “Just Having a Job”. Career Advancement for Low-Wage Workers With Intellectual and Developmental Disabilities. *Career Development and Transition for Exceptional Individuals, 37*(1), 40-49. doi:10.1177/2165143414522092

[122] Ling, T. J., & O'Brien, K. M. (2012). Connecting the Forgotten Half: The School-to-Work Transition of Noncollege-Bound Youth. *Journal of Career Development, 40*(4), 347-367. doi:10.1177/0894845312455506

[123] Lipshits-Braziler, Y., Braunstein-Bercovitz, H., & Kapach-Royf, N. (2018). Strategies for Coping With Career Indecision During the College-to-Work Transition: Concurrent and Predictive Validity. *Journal of Career Assessment, 27*(3), 440-456. doi:10.1177/1069072718759983

[124] López‐Andreu, M., Subramanian, D., & Miquel Verd, J. (2013). Employer strategies, capabilities and career development: two case studies of Spanish service firms. *International Journal of Manpower, 34*(4), 345-361. doi:10.1108/ijm-05-2013-0094

[125] Lounsbury, J. W., Loveland, J. M., Sundstrom, E. D., Gibson, L. W., Drost, A. W., & Hamrick, F. L. (2016). An investigation of personality traits in relation to career satisfaction. *Journal of Career Assessment, 11*(3), 287-307. doi:10.1177/1069072703254501

[126] MacKenzie, R., & Marks, A. (2019). Older workers and occupational identity in the telecommunications industry: Navigating employment transitions through the life course. *Work, Employment and Society, 33*(1), 39-55.

[127] Mallon, M. (1999). Going “portfolio”: Making sense of changing careers. *Career Development International.*

[128] Manderscheid, S., & Harrower, N. L. (2016). A Qualitative study of leader transition and polarities. *Advances in Developing Human Resources, 18*(3), 390-408. doi:10.1177/1523422316645888

[129] Mansuy, M., & Werquin, P. (2018). Moroccan youth and employment: gender differences. *Journal of Education and Work, 31*(5-6), 545-562. doi:10.1080/13639080.2018.1541504

[130] Marin, P. A., & Splete, H. (1991). A Comparison of the effect of two computer‐based counseling interventions on the career decidedness of adults. *The Career Development Quarterly, 39*(4), 360-371.

[131] Martin, H. J., & Lekan, D. F. (2008). Individual differences in outplacement success. *Career Development International, 13*(5), 425-439. doi:10.1108/13620430810891455

[132] Matijaš, M., & Seršić, D. M. (2021). The relationship between career adaptability and job-search self-efficacy of graduates: The bifactor approach. *Journal of Career Assessment, 29*(4), 683-698. doi:10.1177/10690727211002281

[133] McCormick, S. T., Kurth, N. K., Chambless, C. E., Ipsen, C., & Hall, J. P. (2021). Case Management Strategies to Promote Employment for Transition-Age Youth With Disabilities. *Career Development and Transition for Exceptional Individuals*, *44*(2), 120–131. <https://doi.org/10.1177/2165143421991826>

[134] McDonald, P., Pini, B., Bailey, J., & Price, R. (2011). Young people’s aspirations for education, work, family and leisure. *Work, Employment and Society, 25*(1), 68-84. doi:10.1177/0950017010389242

[135] McMahon, M., Watson, M., & Bimrose, J. (2012). Career adaptability: A qualitative understanding from the stories of older women. *Journal of Vocational Behavior, 80*(3), 762-768. doi:10.1016/j.jvb.2012.01.016

[136] Michaeli, Y., Dickson, D. J., & Shulman, S. (2016). Parental and nonparental career-related support among young adults: antecedents and psychosocial correlates. *Journal of Career Development, 45*(2), 150-165. doi:10.1177/0894845316671428

[137] Miner, A. S., & Robinson, D. F. (1994). Organizational and population level learning as engines for career transitions. *Journal of Organizational Behavior, 15*(4), 345-364.

[138] Monteiro, S., & Almeida, L. S. (2015). The relation of career adaptability to work experience, extracurricular activities, and work transition in Portuguese graduate students. *Journal of Vocational Behavior, 91*, 106-112. doi:10.1016/j.jvb.2015.09.006

[139] Mortimer, J. T., Vuolo, M., Staff, J., Wakefield, S., & Xie, W. (2008). Tracing the timing of “career” acquisition in a contemporary youth cohort. *Work and Occupations, 35*(1), 44-84.

[140] Multon, K. D., Wood, R., Heppner, M. J., & Gysbers, N. C. (2016). A cluster-analytic investigation of subtypes of adult career counseling clients: toward a taxonomy of career problems. *Journal of Career Assessment, 15*(1), 66-86. doi:10.1177/1069072706294508

[141] Nabi, G., Holden, R., & Walmsley, A. (2010). From student to entrepreneur: towards a model of graduate entrepreneurial career‐making. *Journal of Education and Work, 23*(5), 389-415. doi:10.1080/13639080.2010.515968

[142] Nery-Kjerfve, T., & Wang, J. (2019). Transfer from expatriate to local contracts: a multiple case study of an unexpected career transition. *Human Resource Development International, 22*(3), 235-256. doi:10.1080/13678868.2019.1570776

[143] Neuenschwander, M. P., & Hofmann, J. (2021). Career decision, work adjustment, and person–job fit of adolescents: moderating effects of parental support. *Journal of Career Development*. doi:10.1177/0894845321995960

[144] Ng, W.-H., Menzies, J., & Zutshi, A. (2019). Facilitators and inhibitors of international postgraduate students’ university-to-work transition. *Australian Journal of Career Development, 28*(3), 186-196. doi:10.1177/1038416219845392

[145] Nicholson, N., & Arnold, J. (1989). Graduate entry and adjustment to corporate life. *Personnel Review, 18*(3), 23-35. doi:10.1108/00483488910133468

[146] Nielsen, M. W. (2016). Reasons for leaving the academy: a case study on the ‘opt out’ phenomenon among younger female researchers. *Gender, Work & Organization, 24*(2), 134-155. doi:10.1111/gwao.12151

[147] Nurmi, J.-E., Salmela-Aro, K., & Koivisto, P. (2002). Goal importance and related achievement beliefs and emotions during the transition from vocational school to work: antecedents and consequences. *Journal of Vocational Behavior, 60*(2), 241-261. doi:10.1006/jvbe.2001.1866

[148] O'connor, D., & Wolfe, D. M. (1991). From crisis to growth at midlife: Changes in personal paradigm. *Journal of Organizational Behavior, 1, 2*(4), 323-340.

[149] Ocampo, A. C. G., Reyes, M. L., Chen, Y., Restubog, S. L. D., Chih, Y.-Y., Chua-Garcia, L., & Guan, P. (2020). The role of internship participation and conscientiousness in developing career adaptability: A five-wave growth mixture model analysis. *Journal of Vocational Behavior, 120*. doi:10.1016/j.jvb.2020.103426

[150] Ogbuanya, T. C., & Chukwuedo, S. O. (2017). Career-training mentorship intervention via the Dreyfus model: Implication for career behaviors and practical skills acquisition in vocational electronic technology. *Journal of Vocational Behavior, 103*, 88-105. doi:10.1016/j.jvb.2017.09.002

[151] Oh, J., Shirmohammadi, M., Jeong, S., & Wang, J. (2021). Leaving the military to work in civilian society: Career adaptability by South Korean short- and mid-term veterans. *Career Development International*, *26*(3), 415–434. <https://doi.org/10.1108/CDI-09-2020-0227>

[152] Ojala, K., Isopahkala-Bouret, U., & Varhelahti, M. (2021). Adult graduates’ employability and mid-career trajectories after graduation with Finnish UAS Master’s degree. *Journal of Education and Work, 34*(1), 67-80. doi:10.1080/13639080.2021.1875125

[153] Okay-Somerville, B., Ricardo Rodrigues, D., David Guest, P., & Scholarios, D. (2014). Coping with career boundaries and boundary-crossing in the graduate labour market. *Career Development International, 19*(6), 668-682. doi:10.1108/cdi-12-2013-0144

[154] Okay-Somerville, B., & Scholarios, D. (2021). Focused for some, exploratory for others: job search strategies and successful university-to-work transitions in the context of labor market ambiguity. *Journal of Career Development*. doi:10.1177/08948453211016058

[155] Okolie, U. C. (2022). Work placement learning and students’ readiness for school-to-work transition: Do perceived employability and faculty supervisor support matter? *Journal of Vocational Behavior*, *139*, 103805. <https://doi.org/10.1016/j.jvb.2022.103805>

[156] Oliveira, M. C. d., Melo-Silva, L. L., Taveira, M. d. C., & Grace, R. C. (2016). Measuring university-to-work success: development of a new scale. *Career Development International, 21*(1), 85-104. doi:10.1108/cdi-04-2015-0051

[157] Oplatka, I. (2001). Self-renewal and inter-organizational transition among women principals. *Journal of Career Development, 28*(1 ), 59-75.

[158] Packard, B. W. L., Leach, M., Ruiz, Y., Nelson, C., & DiCocco, H. (2012). School‐to‐work transition of career and technical education graduates. *The Career Development Quarterly, 60*(2), 134-144.

[159] Pan, J., Guan, Y., Wu, J., Han, L., Zhu, F., Fu, X., & Yu, J. (2018). The interplay of proactive personality and internship quality in Chinese university graduates' job search success: The role of career adaptability. *Journal of Vocational Behavior, 109*, 14-26. doi:10.1016/j.jvb.2018.09.003

[160] Pajic, S., Ulceluse, M., Kismihók, G., Mol, S. T., & den Hartog, D. N. (2018). Antecedents of job search self-efficacy of syrian refugees in greece and the netherlands. Journal of Vocational Behavior, 105, 159-172. doi:10.1016/j.jvb.2017.11.001

[161] Parmentier, M., Pirsoul, T., & Nils, F. (2021). Career adaptability profiles and their relations with emotional and decision-making correlates among Belgian undergraduate students. *Journal of Career Development*. doi:10.1177/08948453211005553

[162] Peeters, E. R., Caniëls, M. C. J., & Verbruggen, M. (2022). Dust yourself off and try again: The positive process of career changes or shocks and career resilience. *Career Development International*, *27*(3), 372–390. <https://doi.org/10.1108/CDI-06-2021-0143>

[163] Perez-Brena, N. J., Wheeler, L. A., Rodriguez De Jesus, S. A., Updegraff, K. A., & Umana-Taylor, A. Y. (2017). The educational and career adjustment of Mexican-origin youth in the context of the 2007/2008 economic recession. *Journal of Vocational Behavior, 100*, 149-163. doi:10.1016/j.jvb.2017.02.006

[164] Pham, Y. K., & Murray, C. (2018). Career locus of control and school- and career-related adjustment among high-need youth with and without disabilities. *Journal of Career Development, 46*(5), 502-515. doi:10.1177/0894845318776801

[165] Pinquart, M., Juang, L. P., & Silbereisen, R. K. (2003). Self-efficacy and successful school-to-work transition: A longitudinal study. *Journal of Vocational Behavior, 63*(3), 329-346. doi:10.1016/s0001-8791(02)00031-3

[166] Pirsoul, T., Parmentier, M., & Nils, F. (2022). Emotional Intelligence Profiles and Job Search Correlates in the Context of the School-to-Work Transition. *Journal of Career Development*, 089484532211414. <https://doi.org/10.1177/08948453221141445>

[167] Popadiuk, N. E., & Arthur, N. M. (2013). Key relationships for international student university-to-work transitions. *Journal of Career Development, 41*(2), 122-140. doi:10.1177/0894845313481851

[168] Presti, A. L., Capone, V., Aversano, A., & Akkermans, J. (2021). Career competencies and career success: on the roles of employability activities and academic satisfaction during the school-to-work transition. *Journal of Career Development*. doi:10.1177/0894845321992536

[169] Raitano, M., & Subioli, F. (2022). School-to-work transition, early career outcomes and income dynamics across cohorts in Italy: Does education pay? *International Journal of Manpower*. <https://doi.org/10.1108/IJM-06-2021-0394>

[170] Ramos, K., & Lopez, F. G. (2018). Attachment security and career adaptability as predictors of subjective well-being among career transitioners. *Journal of Vocational Behavior, 104*, 72-85. doi:10.1016/j.jvb.2017.10.004

[171] Reitzle, M., & Vondracek, F. W. (2000). Methodological avenues for the study of career pathways. *Journal of Vocational Behavior, 57*(3), 445-467. doi:10.1006/jvbe.2000.1751

[172] Renn, R. W., Steinbauer, R., Taylor, R., & Detwiler, D. (2014). School-to-work transition: Mentor career support and student career planning, job search intentions, and self-defeating job search behavior. *Journal of Vocational Behavior, 85*(3), 422-432. doi:10.1016/j.jvb.2014.09.004

[173] Reybold, L. E., & Alamia, J. J. (2008). Academic transitions in education: A developmental perspective of women faculty experiences. Journal of Career Development, 35(2), 107-128. doi:10.1177/0894845308325644

[174] Rigotti, T., Korek, S., & Otto, K. (2014). Gains and losses related to career transitions within organisations. *Journal of Vocational Behavior, 84*(2), 177-187. doi:10.1016/j.jvb.2013.12.006

[175] Robert, P. (2014). Job mismatch in early career of graduates under post-communism. *International Journal of Manpower, 35*(4), 500-513. doi:10.1108/ijm-05-2013-0113

[176] Roberts, K., Teshmatullo, A., Firdavsiy, K., Sarateppo, B., & Tholen, J. (2008). Planned transitions from education into employment in a managed post‐communist market economy: a case study in Samarkand. *Journal of Education and Work, 20*(5), 437-451. doi:10.1080/13639080701814372

[177] Robertson, H. C. (2013). Income and support during transition from a military to civilian career. . *Journal of Employment Counseling, 50*(1), 26-33.

[178] Rose, P. S. (2017). The intern-to-employee career transition. *Journal of Career Development, 45*(6), 566-579. doi:10.1177/0894845317725192

[179] Ruiz, A. C. (2014). Inter-firm job mobility and occupational transitions in spain: Are they related? *Employee Relations, 36*(6), 674-692. doi:10.1108/ER-05-2013-0057

[180] Ruiz-Castro, M., Van der Heijden, B., & Henderson, E. L. (2020). Catalysts in career transitions: Academic researchers transitioning into sustainable careers in data science. *Journal of Vocational Behavior, 122*. doi:10.1016/j.jvb.2020.103479

[181] Rummel, S., Akkermans, J., Blokker, R., & Van Gelderen, M. (2019). Shocks and entrepreneurship: a study of career shocks among newly graduated entrepreneurs. *Career Development International, 26*(4), 562-581. doi:10.1108/cdi-11-2018-0296

[182] Ruschoff, B., Kowalewski, T., & Salmela-Aro, K. (2021). The effects of peers’ career goal appraisals on school to work transition outcomes. *Journal of Career Development*. doi:10.1177/08948453211020132

[183] Ruschoff, B., Salmela-Aro, K., Kowalewski, T., Dijkstra, J. K., & Veenstra, R. (2018). Peer networks in the school-to-work transition. *Career Development International, 23*(5), 466-477. doi:10.1108/cdi-02-2018-0052

[184] Ryan, M. J., & Chambers, T. P. (2015). An exploration of career decision-making in the Australian Football League: Does playing experience matter? *Australian Journal of Career Development, 24*(2), 114-119. doi:10.1177/1038416215584407

[185] Samaluk, B. (2020). Precarious education-to-work transitions: Entering welfare professions under a workfarist regime. *Work, Employment and Society, 35*(1), 137-156. doi:10.1177/0950017020931335

[186] Santilli, S., Nota, L., & Hartung, P. J. (2019). Efficacy of a group career construction intervention with early adolescent youth. *Journal of Vocational Behavior, 111*, 49-58. doi:10.1016/j.jvb.2018.06.007

[187] Schoon, I., Martin, P., & Ross, A. (2007). Career transitions in times of social change. His and her story. *Journal of Vocational Behavior, 70*(1), 78-96. doi:10.1016/j.jvb.2006.04.009

[189] Scott, C. M. (2002). Counseling adults in career transition: Reflections of a counselor-in-training. *Journal of Career Development, 28*(3 ), 215-220.

[190] Sedge, S. K. ((1985). A comparison of engineers pursuing alternate career paths. *Journal of Vocational Behavior, 27*(1), 56-70.

[191] Shearer, C. B. (2009). Exploring the relationship between intrapersonal intelligence and university students' career confusion: implications for counseling, academic success, and school‐to‐career transition. *Journal of Employment Counseling, 46*(2), 52-61.

[192] Shen, Y., & Kram, K. E. (2011). Expatriates' developmental networks: network diversity, base, and support functions. *Career Development International, 16*(6), 528-552. doi:10.1108/13620431111178317

[193] Shu, S., Wang, Y., Kang, H., Wu, C.-H., & Arenius, P. (2023). Understanding the role of job quality in the association of employees’ career change to self-employment and job satisfaction. *Personnel Review*, *52*(1), 288–303. <https://doi.org/10.1108/PR-03-2021-0212>

[194] Singh, G., Saghafi, M., Ehrlich, S., & De Noble, A. (2010). Perceptions of self-employment among mid-career executives in the People’s Republic of China. *Journal of Career Assessment, 18*(4), 393-408. doi:10.1177/1069072710374579

[195] Sortheix, F. M., Chow, A., & Salmela-Aro, K. (2015). Work values and the transition to work life: A longitudinal study. *Journal of Vocational Behavior, 89*, 162-171. doi:10.1016/j.jvb.2015.06.001

[196] Sortheix, F. M., Dietrich, J., Chow, A., & Salmela-Aro, K. (2013). The role of career values for work engagement during the transition to working life. *Journal of Vocational Behavior, 83*(3), 466-475. doi:10.1016/j.jvb.2013.07.003

[197] Srinivas, S. (2009). The impact of technological mobility on workers' careers. *Career Development International, 14*(2), 133-147. doi:10.1108/13620430910950746

[198] Steiner, R. S., Hirschi, A., & Wang, M. (2019). Predictors of a protean career orientation and vocational training enrollment in the post-school transition. *Journal of Vocational Behavior, 112*, 216-228. doi:10.1016/j.jvb.2019.03.002

[199] Sterrett, E. A. (1999). A comparison of women's and men's career transitions. *Journal of Career Development, 25*(4), 249-259.

[200] Stiwne, E. E., & Jungert, T. (2010). Engineering students’ experiences of transition from study to work. *Journal of Education and Work, 23*(5), 417-437. doi:10.1080/13639080.2010.515967

[201] Stoltz, K. B., Wolff, L. A., Monroe, A. E., Farris, H. R., & Mazahreh, L. G. (2013). Adlerian lifestyle, stress coping, and career adaptability: Relationships and dimensions. *The Career Development Quarterly, 61*(3), 194-209. doi:10.1002/j.2161-0045.2013.00049.x

[202] Stout, S. K., Slocum Jr, J. W., & Cron, W. L. (1987). Career transitions of superiors and subordinates. *Journal of Vocational Behavior, 30*(2), 124-137.

[203] Stringer, K., Kerpelman, J., & Skorikov, V. (2011). Career preparation: A longitudinal, process-oriented examination. *Journal of Vocational Behavior, 79*(1), 158-169. doi:10.1016/j.jvb.2010.12.012

[204] Sundstrom, E. D., Lounsbury, J. W., Gibson, L. W., & Huang, J. L. (2016). Personality Traits and Career Satisfaction in Training and Development Occupations: Toward a Distinctive T&D Personality Profile. *Human Resource Development Quarterly, 27*(1), 13-40. doi:10.1002/hrdq.21223

[205] Šverko, I., & Babarović, T. (2019). Applying career construction model of adaptation to career transition in adolescence: A two-study paper. *Journal of Vocational Behavior, 111*, 59-73. doi:10.1016/j.jvb.2018.10.011

[206] Takeuchi, T., Takeuchi, N., & Jung, Y. (2020). Toward a process model of newcomer socialization: Integrating pre‐ and post‐entry factors for newcomer adjustment. *Human Resource Development Quarterly, 32*(3), 391-418. doi:10.1002/hrdq.21420

[207] Taylor, A., & Servage, L. (2012). Perpetuating education–jobs mismatch in a high school internship programme: an ecological model. *Journal of Education and Work, 25*(2), 163-183. doi:10.1080/13639080.2011.565041

[208] Terblanche, N. (2020). Coaching techniques for sustained individual change during career transitions. *Human Resource Development Quarterly, 32*(1), 11-33. doi:10.1002/hrdq.21405

[209] Terjesen, S., & Burke, R. (2005). Senior women managers’ transition to entrepreneurship. *Career Development International, 10*(3), 246-259. doi:10.1108/13620430510598355

[210] Terjesen, S., & Sullivan, S. E. (2011). The role of developmental relationships in the transition to entrepreneurship. *Career Development International, 16*(5), 482-506. doi:10.1108/13620431111168895

[211] Thompson, J. A., & Van de Ven, A. H. (2002). Commitment shift during organizational upheaval: Physicians' transitions from private practitioner to employee. *Journal of Vocational Behavior, 60*(3), 382-404.

[212] Tolentino, L. R., Sibunruang, H., & Garcia, P. R. J. M. (2018). The role of self-monitoring and academic effort in students’ career adaptability and job search self-efficacy. *Journal of Career Assessment, 27*(4), 726-740. doi:10.1177/1069072718816715

[213] Tomlinson, M. (2007). Graduate employability and student attitudes and orientations to the labour market. *Journal of Education and Work, 20*(4), 285-304. doi:10.1080/13639080701650164

[214] Topa, G., & Alcover, C.-M. (2015). Psychosocial factors in retirement intentions and adjustment: a multi-sample study. *Career Development International, 20*(4), 384-408. doi:10.1108/cdi-09-2014-0129

[215] Toyokawa, T., & DeWald, C. (2020). Perceived career barriers and career decidedness of first‐generation college students. *The Career Development Quarterly, 68*(4), 332-347. doi:10.1002/cdq.12240

[216] Tran, T. T. (2014). Is graduate employability the ‘whole-of-higher-education-issue’? *Journal of Education and Work, 28*(3), 207-227. doi:10.1080/13639080.2014.900167

[217] Tynkkynen, L., Nurmi, J.-E., & Salmela-Aro, K. (2010). Career goal-related social ties during two educational transitions: Antecedents and consequences. *Journal of Vocational Behavior, 76*(3), 448-457. doi:10.1016/j.jvb.2009.12.001

[218] Ulrich, L. B., & Brott, P. E. (2005). Older workers and bridge employment: Redefining retirement. . *Journal of Employment Counseling, 42*(4 ), 159-170.

[219] Umney, C., & Kretsos, L. (2015). “That’s the experience”: Passion, work precarity, and life transitions among London jazz musicians. *Work and Occupations, 42*(3), 313-334. doi:10.1177/0730888415573634

[220] Upadyaya, K., & Salmela-Aro, K. (2015). Development of early vocational behavior: Parallel associations between career engagement and satisfaction. *Journal of Vocational Behavior, 90*, 66-74. doi:10.1016/j.jvb.2015.07.008

[221] Van der Horst, A. C., Klehe, U.-C., Brenninkmeijer, V., & Coolen, A. C. M. (2021). Facilitating a successful school-to-work transition: Comparing compact career-adaptation interventions. *Journal of Vocational Behavior, 128*. doi:10.1016/j.jvb.2021.103581

[222] Van der Horst, A. C., Klehe, U.-C., & Van der Heijden, B. I. J. M. (2017). Adapting to a looming career transition: How age and core individual differences interact. *Journal of Vocational Behavior, 99*, 132-145. doi:10.1016/j.jvb.2016.12.006

[223] Vaughan, K., & Roberts, J. (2007). Developing a ‘productive’ account of young people’s transition perspectives. *Journal of Education and Work, 20*(2), 91-105. doi:10.1080/13639080701314621

[224] Vincent, C., Glow H., Johanson K., Coate B. (2021). Who did you meet at the Venice Biennale? education- to-work transition enhancers for aspiring arts professionals in Australia. *Work, Employment and Society, 1*(18).

[225] Vogtenhuber, S. (2014). The impact of within country heterogeneity in vocational specificity on initial job matches and job status. *Journal of Vocational Behavior, 85*(3), 374-384. doi:10.1016/j.jvb.2014.08.012

[226] Vuori, J., Koivisto, P., Mutanen, P., Jokisaari, M., & Salmela-Aro, K. (2008). Towards Working Life: Effects of an intervention on mental health and transition to post-basic education. *Journal of Vocational Behavior, 72*(1), 67-80. doi:10.1016/j.jvb.2007.10.003

[227] Wang, C., Lo, Y.-y., Xu, Y., Wang, Y., & Porfeli, E. (2007). Constructing the search for a job in academia from the perspectives of self-regulated learning strategies and social cognitive career theory. *Journal of Vocational Behavior, 70*(3), 574-589. doi:10.1016/j.jvb.2007.02.002

[228] Waters, L., Briscoe, J. P., Hall, D. T., & Wang, L. (2014). Protean career attitudes during unemployment and reemployment: A longitudinal perspective. *Journal of Vocational Behavior, 84*(3), 405-419. doi:10.1016/j.jvb.2014.03.003

[229] Wiesner, M., Vondracek, F. W., Capaldi, D. M., & Porfeli, E. (2003). Childhood and adolescent predictors of early adult career pathways. *Journal of Vocational Behavior, 63*(3), 305-328. doi:10.1016/s0001-8791(03)00028-9

[230] Wilckens, M. R., Wöhrmann, A. M., Deller, J., & Finsel, J. (2022). Health and the intention to retire: Exploring the moderating effects of human resources practices. *The International Journal of Human Resource Management*, 1–35. <https://doi.org/10.1080/09585192.2022.2133967>

[231] Winterheller, J., & Hirt, C. (2017). Career patterns of young highly skilled migrants from Southeast Europe in Austria. *Personnel Review, 46*(2), 222-236. doi:10.1108/pr-05-2015-0148

[232] Wordsworth, R., & Nilakant, V. (2021). Unexpected change: Career transitions following a significant extra-organizational shock. *Journal of Vocational Behavior, 127*. doi:10.1016/j.jvb.2021.103555

[233] Xu, Y. J. (2016). Attrition of Women in STEM. *Journal of Career Development, 44*(1), 3-19. doi:10.1177/0894845316633787

[234] Yakushko, O., & Sokolova, O. (2010). Work hope and influences of the career development among Ukrainian college students. *Journal of Career Development, 36*(4), 310-323. doi:10.1177/0894845309345670

[235] Yang, E., & Gysbers, N. C. (2007). Career transitions of college seniors. *The Career Development Quarterly, 56*(2), 157-170.

[236] Zaniboni, S., Shultz, K., Sarchielli, G., & Fraccaroli, F. (2010). How are psychosocial factors related to retirement intentions? *International Journal of Manpower, 31*(3), 271-285. doi:10.1108/01437721011050576

[237] Zhang, C., Hirschi, A., Li, M., & You, X. (2021). Profiles of calling and their relation to university-to-work transition outcomes. *Journal of Career Development*. doi:10.1177/0894845321992873

[238] Zhang, W., Guan, X., Zhou, X., & Lu, J. (2019). The effect of career adaptability on career planning in reaction to automation technology. *Career Development International, 24*(6), 545-559. doi:10.1108/cdi-05-2018-0135

[239] Zhang, Y., Wang, Q., Zhang, Y., Xu, C., & Xu, Z. (2022). Protean Career Orientation and Proactive Career Behaviors During School-to-Work Transition: Mechanism Exploration and Coaching Intervention. *Journal of Career Development*, 089484532211135. <https://doi.org/10.1177/08948453221113545>

[240] Zhou, Y., Wu, C. H., Zou, M., & Williams, M. (2021). When is the grass greener on the other side? A longitudinal study of the joint effect of occupational mobility and personality on the honeymoon‐hangover experience during job change. *Journal of Organizational Behavior, 42*(4), 551-566. doi:10.1002/job.2491

[241] Zikic, J., Bonache, J., & Cerdin, J.-L. (2010). Crossing national boundaries: A typology of qualified immigrants' career orientations. *Journal of Organizational Behavior, 31*(5), 667-686. doi:10.1002/job.705
